# Calcineurin Targets Involved in Stress Survival and Fungal Virulence

**DOI:** 10.1371/journal.ppat.1005873

**Published:** 2016-09-09

**Authors:** Hee-Soo Park, Eve W. L. Chow, Ci Fu, Erik J. Soderblom, M. Arthur Moseley, Joseph Heitman, Maria E. Cardenas

**Affiliations:** 1 Department of Molecular Genetics and Microbiology, Duke University Medical Center, Durham, North Carolina, United States of America; 2 Duke Proteomics and Metabolomics Core Facility, Center for Genomic and Computational Biology, Duke University, Durham, North Carolina, United States of America; Carnegie Mellon University, UNITED STATES

## Abstract

Calcineurin governs stress survival, sexual differentiation, and virulence of the human fungal pathogen *Cryptococcus neoformans*. Calcineurin is activated by increased Ca^2+^ levels caused by stress, and transduces signals by dephosphorylating protein substrates. Herein, we identified and characterized calcineurin substrates in *C*. *neoformans* by employing phosphoproteomic TiO_2_ enrichment and quantitative mass spectrometry. The identified targets include the transactivator Crz1 as well as novel substrates whose functions are linked to P-bodies/stress granules (PBs/SGs) and mRNA translation and decay, such as Pbp1 and Puf4. We show that Crz1 is a *bona fide* calcineurin substrate, and Crz1 localization and transcriptional activity are controlled by calcineurin. We previously demonstrated that thermal and other stresses trigger calcineurin localization to PBs/SGs. Several calcineurin targets localized to PBs/SGs, including Puf4 and Pbp1, contribute to stress resistance and virulence individually or in conjunction with Crz1. Moreover, Pbp1 is also required for sexual development. Genetic epistasis analysis revealed that Crz1 and the novel targets Lhp1, Puf4, and Pbp1 function in a branched calcineurin pathway that orchestrates stress survival and virulence. These findings support a model whereby calcineurin controls stress and virulence, at the transcriptional level via Crz1, and post-transcriptionally by localizing to PBs/SGs and acting on targets involved in mRNA metabolism. The calcineurin targets identified in this study share little overlap with known calcineurin substrates, with the exception of Crz1. In particular, the mRNA binding proteins and PBs/SGs residents comprise a cohort of novel calcineurin targets that have not been previously linked to calcineurin in mammals or in *Saccharomyces cerevisiae*. This study suggests either extensive evolutionary rewiring of the calcineurin pathway, or alternatively that these novel calcineurin targets have yet to be characterized as calcineurin targets in other organisms. These findings further highlight *C*. *neoformans* as an outstanding model to define calcineurin-responsive virulence networks as targets for antifungal therapy.

## Author Summary

Calcineurin is a Ca^2+^/calmodulin-dependent protein phosphatase essential for stress survival, sexual development, and virulence of the human fungal pathogen *Cryptococcus neoformans* and other major pathogenic fungi of global human health relevance. However, no calcineurin substrates are known in pathogenic fungi. Employing state-of-the-art phosphoproteomic approaches we identified calcineurin substrates, including calcineurin itself and the conserved Crz1 transcriptional activator known to function in calcium signaling and stress survival. Remarkably, our study also identified novel calcineurin targets involved in RNA processing, stability, and translation, which colocalize together with calcineurin in stress granules/P-bodies upon thermal stress. These findings support a model whereby calcineurin functions in a branched pathway, via Crz1 and several of the identified novel targets, that governs transcriptional and posttranscriptional circuits to drive stress survival, sexual development, and fungal virulence. Our study underscores *C*. *neoformans* as an experimental model to define basic paradigms of calcineurin signaling in global thermostress responsive virulence networks that can be targeted for fungal therapy.

## Introduction

*Cryptococcus neoformans* is an environmental fungus found ubiquitously throughout the world [1,2]. Both spores and desiccated yeast cells are infectious propagules and exposure via inhalation causes life-threatening disease [2,3]. *C*. *neoformans* is primarily an opportunistic pathogenic fungus that causes meningoencephalitis, frequently in immunocompromised patients with HIV/AIDS, organ transplants, or autoimmune diseases [4,5]. Key virulence attributes of *Cryptococcus* are its ability to adapt to stressful host environments, including the elevated body temperature of the mammalian host [6,7].

Calcineurin is necessary for *Cryptococcus* to effectively survive host thermal stress [8]. Calcineurin is a virulence factor conserved in human fungal pathogens across species including *C*. *neoformans*, *Candida albicans*, *Aspergillus fumigatus*, and *Mucor circinelloides* [8–12]. Two immunosuppressive natural products, FK506 and cyclosporin A (CsA), bind to intracellular proteins, cyclophilin A and FKBP12 respectively, and the protein-drug complexes then inhibit calcineurin [13–17]. Because calcineurin is conserved from fungi to humans, FK506 and CsA exhibit broad antifungal and immunosuppressive activities [18–21].

Calcineurin is a Ca^2+^/calmodulin-activated serine/threonine-specific protein phosphatase consisting of two subunits: a catalytic A subunit and a regulatory B subunit [22,23]. In response to internal or external stress-derived signals intracellular Ca^2+^ levels increase, Ca^2+^ binds to calmodulin, and Ca^2+^-calmodulin then binds to the catalytic A subunit of calcineurin, leading to calcineurin activation [24]. Activated calcineurin dephosphorylates target proteins, which in turn modulate various biological processes [25]. In mammals such as mice and humans, calcineurin dephosphorylates the NFAT (nuclear factor of activated T cell) family of transcription factors that controls transcription of genes required for T cell activation, cardiac hypertrophy, and development [26–28]. In the model budding yeast *Saccharomyces cerevisiae*, Crz1 (calcineurin-responsive zinc finger 1) was identified as a calcineurin-activated transcription factor inducing transcription of genes involved in stress responses [29–32]. Recent studies identified Crz1 orthologues in multiple ascomycetous fungal species and other eukaryotes [31]. Recently, three groups characterized a candidate Crz1/Sp1 transcription factor in the human pathogenic fungus *C*. *neoformans* and demonstrated that it plays crucial roles in cell wall integrity and virulence; however, only one of these studies concluded that this was a *bona fide* Crz1 ortholog [33–35].

Calcineurin is required for growth at 37°C, virulence, and sexual reproduction in the fungal pathogen *C*. *neoformans* [9,36,37]. Our previous studies found that calcineurin is re-localized from the cytoplasm to puncta and the mother-bud neck in response to heat and other stresses [38,39]. Calcineurin was found to co-localize in puncta with components of PBs/SGs, which are known to contain non-translating mRNPs (messenger ribonucleoprotein complexes) [40–42]. These cytoplasmic structures consist of mRNAs associated with translation initiation factors, RNA binding proteins, and the mRNA decay machinery, and function to control translation initiation, mRNA degradation, and siRNA function [43–45]. These findings lead to the hypothesis that PBs/SGs may contain putative calcineurin targets whose functions are governed by calcineurin to promote thermal stress survival.

To identify calcineurin targets, we compared the phosphopeptide profiles obtained from calcineurin-activated (WT cells exposed to 37°C) and calcineurin-deficient conditions (*cna1*Δ mutant or WT cells exposed to FK506). In total, 56 calcineurin-dependent dephosphorylation targets were identified, including Cna1, the Crz1 ortholog, and proteins whose orthologs have roles in stress responses, mRNA binding/stability, protein translation, and vesicular trafficking. Intriguingly, several proteins, including Pbp1 (Poly(A) binding protein-Binding Protein) and Puf4 (PUmilio-homology domain Family) were identified as calcineurin targets and upon exposure to 37°C, co-localize in PBs/SGs with calcineurin. We demonstrate that Crz1 is a *bona fide* calcineurin substrate whose localization and transcriptional activity is controlled by calcineurin, while Pbp1 functions as a calcineurin target involved in calcineurin-dependent sexual reproduction. Moreover, mutation of the genes encoding the calcineurin targets Crz1, Puf4, and Lhp1 conferred hypersensitivity to thermal and other stresses. Epistasis analysis revealed that Crz1 and the RNA binding proteins Puf4, Pbp1, and Lhp1 function in a branched pathway controlled by calcineurin. These results support a model in which calcineurin governs growth at high temperature, virulence, and sexual reproduction by controlling both DNA- and RNA-binding proteins in transcriptional and post-transcriptional circuits.

## Results

### Identification of calcineurin targets via phospho-proteomic analyses

Calcineurin is essential for survival at 37°C and virulence; however, no calcineurin targets have been identified in *C*. *neoformans*. We sought to identify calcineurin targets by employing a quantitative phosphoproteomic approach comprising TiO_2_-based affinity chromatography enrichment of phosphopeptides and quantitative analysis using 2-dimentional ultraperformance liquid chromatography coupled to high-resolution accurate-mass spectrometry (2D-LC/LC-MS/MS). To identify calcineurin-dependent dephosphorylation events at 37°C, we performed three differential expression phosphoproteomic screens. In the first and second screens, we aligned and compared phosphopeptide profiles from WT cells grown at 25°C and shifted to 37°C for 1 hour and either exposed or not to FK506 15 minutes prior to and during the shift to 37°C. For the third screen, phosphopeptide patterns of *cna1*Δ mutant cells grown at 25°C and shifted to 37°C for 1 hr were compared to similarly grown and treated WT cells.

**Fig 1:**
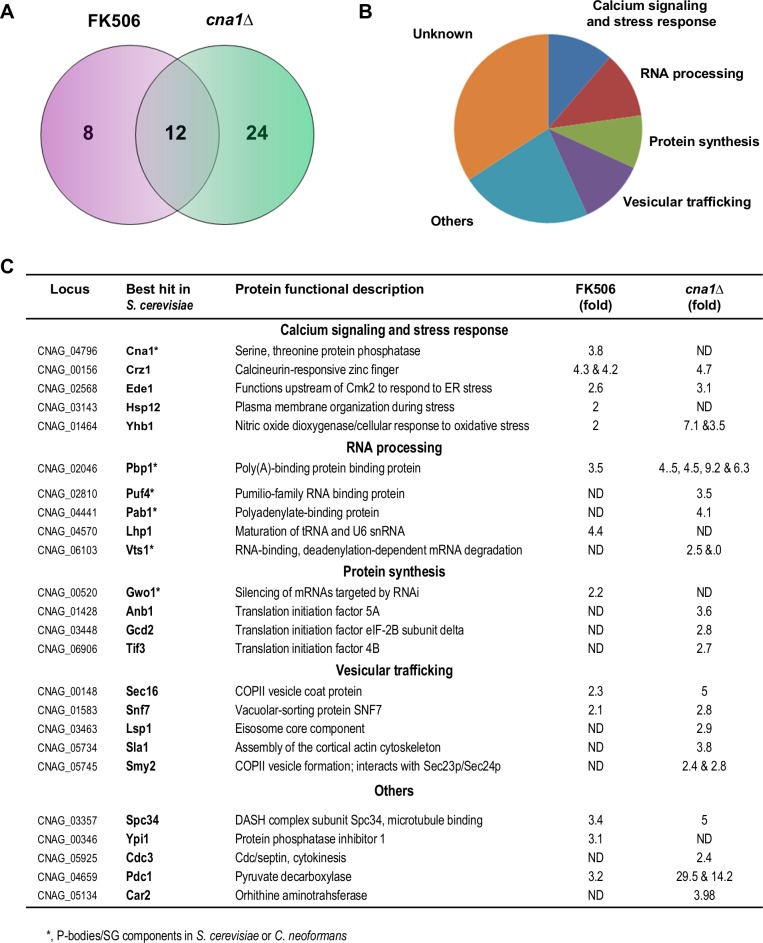
Identification of calcineurin-dependent targets. **(A)** Venn diagram illustrating the number of potential calcineurin targets identified by the phosphopeptide screens and target overlap between the 44 phosphorylated proteins enriched by at least 2-fold in the two calcineurin-deficient samples (FK506 exposure or *cna1* mutation). Phosphopeptide profiles from WT (H99) cells grown at 25°C and shifted to 37°C for 1 hr and exposed or not to FK506 were aligned and compared (FK506). A similar comparison was performed for phosphopeptides profiles from the WT (strain H99) culture shifted from 25°C to 37°C and a *cna1*Δ (strain KK1) culture subjected to the same temperature shift (*cna1Δ*). Biological cultures were performed in duplicate for each condition tested and following trypsin digestion each sample was divided into three to access TiO2 enrichment and analytical reproducibility. **(B)** Functional category of the forty-four putative calcineurin targets. **(C)** Summary of potential calcineurin targets identified at 37°C. Note that this table only lists a subset of the potential calcineurin targets at 37°C with at least a 2-fold or greater signal difference (T-test p-value <0.05) in the two different screens and for which a characterized *S*. *cerevisiae* ortholog is known (based on best hit blast searches against the *S*. *cerevisiae* genome database) and does not include the identified but uncharacterized ORFs. The differential fold change signal for each individual phosphopeptide (see **S2** and **S3 Tables** for detailed and complete individual phosphopeptide data) within the same protein observed under the FK506 or *cna1*Δ condition is indicated. * denotes proteins characterized as components of PB/SGs in *C*. *neoformans*.

In summary, these screens identified 2,016 total peptides (1,398 unique phosphorylated peptides) and 796 total proteins (576 unique phosphorylated proteins) across all samples **(S1 Table)**. We performed a T-test analysis on log2 transformed phosphopeptide intensities in the datasets of the different conditions compared in the screens. Phosphopeptides that were overrepresented by more than 2-fold (T-test p-value <0.05) in the calcineurin-deficient conditions relative to the calcineurin-activated condition were considered as potential calcineurin targets **(S2 and S3 Tables)**. A total of 59 phosphorylated peptides (44 proteins) were more abundant in calcineurin-deficient cells **(S4 and S5 Tables)**. We submitted these 44 ORFs to BLAST searches for functional domain homologies against protein databases and best fits were assigned based on the *S*. *cerevisiae* genome database (SGD). Importantly, we found that only 12 proteins containing phosphopeptides that changed in abundance in calcineurin-deficient strains overlapped between the two different conditions (*cna1*Δ mutant or WT cells exposed to FK506) **(Fig 1A, S5 Table)**. Moreover, more potential calcineurin targets were identified when calcineurin was blocked by mutation (36 targets) than by inhibition with FK506 (20 targets). We interpreted these finding as being the result of a more profound physiological remodeling triggered by permanent calcineurin inhibition in the *cna1* mutant as opposed to the potentially less severe effects imposed by transient inhibition of calcineurin with FK506 **(Fig 1A, S5 Table)**. Similar results were found by a study that identified the calcineurin substrates in *S*. *cerevisiae* [46]. The identified functional categories included proteins involved in calcium signaling or stress responses such as calcineurin A (Cna1) itself, and an ortholog of the transcription factor Crz1 **(Fig 1B and 1C)**. Intriguingly, the screen also identified several proteins whose *S*. *cerevisiae* orthologs are localized to P-bodies and stress granules, including Pbp1, Pab1, Puf4, Vts1, and Gwo1 (Gis2 ortholog) [47–49] as well as other components whose orthologs have functions in protein synthesis and mRNA binding/stability. Another significant functional category that was identified involves vesicular trafficking, in accord with our previous studies that COPI and COPlI members interact and co-localize with calcineurin in ER-associated stress granules [38].

Although it is generally accepted that protein phosphatases do not dephosphorylate specific amino acid sequence motifs, we analyzed the 59 calcineurin-dependent phosphopeptide sequences employing the PhosphoSitePlus software [50] to test if they exhibit any characteristic features. Interestingly, the calcineurin-dependent phosphopeptide containing a phosphoserine residue showed a significant enrichment of Pro at the +1 position or Arg at the -3 position (27 or 16 phosphopeptides out of 47, respectively) with 7 featuring both Arg at -3 and Pro at +1 **(S1A Fig and S5 Table)**. However, the calcineurin-dependent phosphopeptides containing a phosphothreonine residue exhibited a weak potential signature in that 3 or 2 out of 10 had an Arg at the -2 or -3 position, respectively **(S1B Fig and S5 Table)**.

Previous studies demonstrated that calcineurin recognizes specific substrate docking sites such as PxIxIT motifs [51]. To examine if the potential calcineurin substrates identified by our screens contain PxIxIT motifs, we searched for the verified PxIxIT motif (P[^PG][IVLF][^PG][IVLF][TSHEDQNKR]) characterized in *S*. *cerevisiae* [46]. In *C*. *neoformans*, 2,530 predicted ORFs contain the PxIxIT motif. Among the 44 putative calcineurin targets, 13 of them, including Cna1, Crz1, Puf4, and Gcd2 feature a PxIxIT motif **(S5 Table)**. In particular, the Crz1 ortholog contains two potential PxIxIT motifs (PALSIS and PMICIQ), suggesting that Crz1 could represent an authentic calcineurin substrate in *C*. *neoformans*. However, the PxIxIT motif is poorly conserved amongst different fungal species (46) and occurs rather frequently (2536 out of a predicted total 6957 proteins or 36%) in the *Cryptococcus* proteome. Thus, it remains to be tested whether the identified PxlxlT motifs in fact function as calcineurin docking sites in *Cryptococcus*.

### Crz1 is a *bona fide* calcineurin substrate

**Fig 2:**
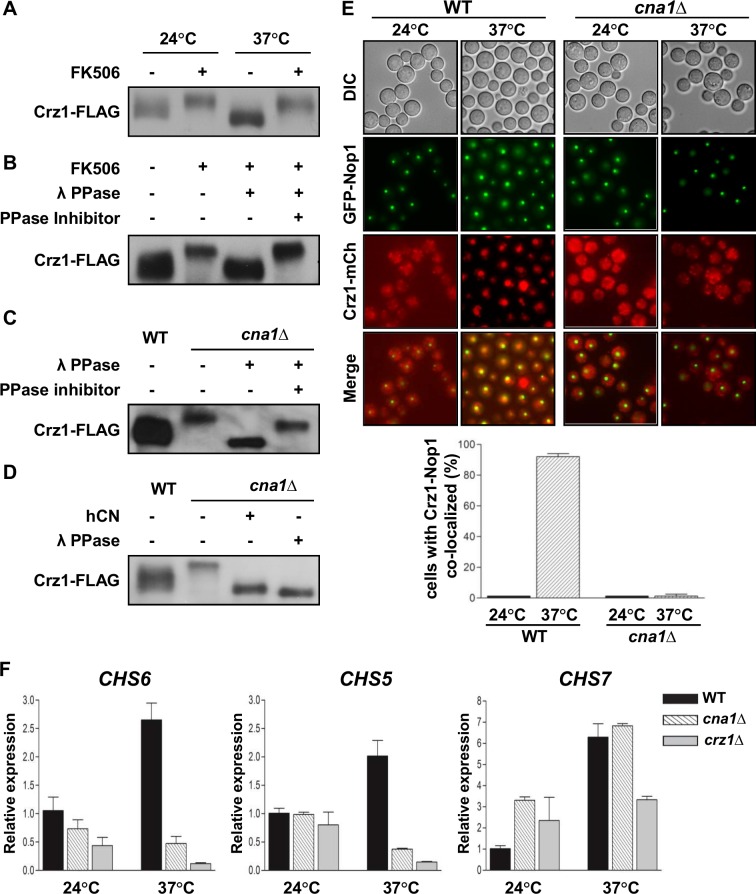
Calcineurin regulates the phosphorylation of Crz1. **(A)** Crz1 electrophoretic mobility is altered by conditions that inhibit or activate calcineurin. Cultures of the *crz1*Δ *CRZ1-*4xFLAG complemented strain (SEC435) were grown to log phase at 24°C or shifted from 24°C to 37°C for 1 hour in the presence or absence of 1 μg/ml FK506. **(B)** Crz1 is a phosphoprotein. The Crz1-4xFLAG protein, immunoprecipitated from logarithmically growing cultures of strain SEC435 (see panel A) exposed or not to 1 μg/ml FK506 for 1 hr, was treated with λ phosphatase in the presence or absence of phosphatase inhibitors. **(C)** The Crz1-FLAG protein was immunoprecipitated from whole cell extracts of the *crz1*Δ + *CRZ1-*4xFLAG complemented strain (SEC435) or the *crz1*Δ + *CRZ1-*4xFLAG *cna1*Δ (HP289) strain. Immunoprecipitated Crz1-FLAG protein was treated with λ phosphatase, with and without phosphatase inhibitors. **(D)** Human calcineurin dephosphorylates Crz1 *in vitro*. Immunopurified Crz1-4xFLAG protein from the *crz1*Δ + *CRZ1-*4xFLAG complemented strain (SEC435) or the *crz1*Δ + *CRZ1-*4xFLAG *cna1*Δ (HP289) strain was treated with human calcineurin or λ phosphatase *in vitro*. In **Fig 2 A-D** following the different treatments, samples were resolved by SDS-PAGE and Crz1-4xFLAG mobility was assessed by western blot analysis as indicated in material methods. **(E)** Localization of Crz1-mCherry in WT (XW252) and the *cna1*Δ (HP282) mutant at 24°C and 37°C (upper panel). Each strain culture was grown to log phase at 24°C or shifted from 24°C to 37°C for 1 hr and visualized with a DeltaVision Elite Deconvolution microscope. The graph represents quantification of cells in which Crz1-mCherry and GFP-Nop1 were co-localized (bottom panel). **(F)** Expression of chitin synthase genes including *CHS5*, *CHS6*, and *CHS7* in WT (H99), *cna1*Δ (KK1), and *crz1*Δ (LK343) strains. WT, *cna1*Δ, and *crz1*Δ strains were grown to log phase at 24°C or shifted from 24°C to 37°C for 1 hr. RNA was isolated from the indicated strains and the expression of the chitin synthase genes was assessed by real-time PCR. Error bars depict the standard deviations from the mean of three independent experiments. Results shown in **Fig 2 A-D** and **2F** are representative of two independent experimental replicates.

To confirm the effectiveness of our phosphoproteomic approach, we first tested if Crz1 is a calcineurin substrate. Cells expressing a C-terminally FLAG-tagged Crz1 protein were grown at 24°C and shifted from 24°C to 37°C for 1 hour in the presence or absence of FK506. The Crz1-FLAG tagged protein exhibited increased electrophoretic mobility when isolated from cells shifted from 24°C to 37°C as compared to that isolated from the culture grown at 24°C **(Fig 2A)**. In contrast, Crz1-FLAG displayed reduced mobility in cells treated with FK506 at both temperatures **(Fig 2A).** To test if the reduction in mobility of Crz1-FLAG is caused by phosphorylation, we immune-isolated the Crz1-FLAG protein from WT cells exposed to FK506, and the isolated protein was incubated with λ phosphatase in the presence or absence of phosphatase inhibitor. The reduced mobility of the Crz1-FLAG protein in FK506 was reversed by treatment with λ phosphatase but not with λ phosphatase plus a phosphatase inhibitor **(Fig 2B)**. Moreover, the mobility pattern of Crz1-FLAG in cells exposed to FK506 was similar to the mobility of Crz1-FLAG when expressed in a *cna1* mutant background **(Fig 2C)**.

We next tested if calcineurin dephosphorylates Crz1 *in vitro*. The immunopurified Crz1-FLAG protein was isolated from WT and *cna1*Δ strains and treated with λ phosphatase or human calcineurin. The Crz1-FLAG protein from the *cna1*Δ strain exhibited decreased mobility compared with the protein from the WT strain and this decreased mobility was reversed by treatment with either human calcineurin or λ phosphatase **(Fig 2D)**. These results demonstrate that Crz1 is a phosphoprotein and a *bona fide* substrate of calcineurin and support the validity of our phosphoproteomic approach.

Previous studies have concluded based on indirect evidence that calcineurin dephosphorylates Crz1 and that this triggers Crz1 translocation into the nucleus in several fungi [31,52,53]. In *C*. *neoformans*, Lev *et al*. presented findings that Crz1 is nuclear localized in response to several stresses, and that this relocalization activates the transcriptional activity of Crz1 [33]. To verify the subcellular localization of Crz1, we examined localization of Crz1-mCherry in WT and *cna1*Δ strains co-expressing the nucleolar marker GFP-Nop1 [54]. The Crz1-mCherry protein was distributed throughout the cytosol in cells grown at room temperature. Crz1 translocated into the nucleus upon calcineurin activation by exposure to 37°C. In contrast, Crz1-mCherry failed to translocate to the nucleus in *cna1*Δ mutant cells in response to temperature shift **(Fig 2E)**.

The chitin synthase genes are known targets of the calcineurin-Crz1 cascade in several fungi [33,55–57]. We therefore examined expression of three chitin synthase genes (*CHS5*, *CHS6*, and *CHS7*) in the WT, and the *cna1*Δ, and *crz1*Δ mutant strains at 24°C and 37°C. Expression of all three chitin synthase genes was induced upon shift from 24°C to 37°C **(Fig 2F)**. Importantly, the induced mRNA expression of *CHS5* and *CHS6*, but not *CHS7*, was prevented by the *cna1*Δ or *crz1*Δ mutation, confirming that expression of the *CHS5* and *CHS6* genes is controlled by the calcineurin-Crz1 pathway. Taken together, these results demonstrate that calcineurin directly dephosphorylates Crz1 and thereby controls Crz1 sub-cellular localization and transcriptional activity in response to elevated temperature.

**Fig 3:**
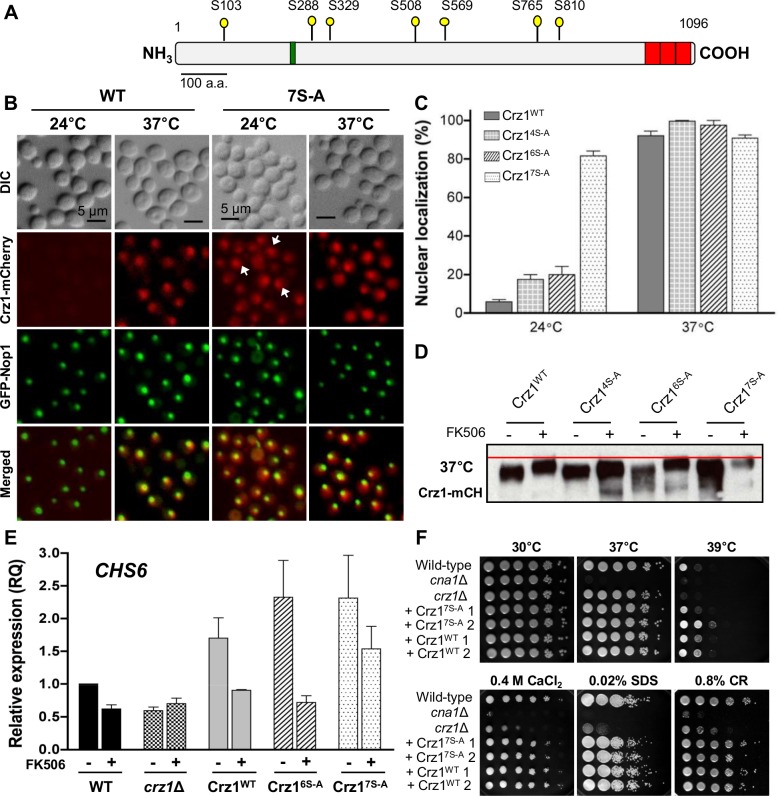
Mutations of calcineurin-dependent phosphorylation sites promotes Crz1 nuclear localization and transcriptional activity at 24°C. **(A)** Schematic diagram of the Crz1 protein drawn to scale. The yellow pins indicate the locations of 7 of the 12 predicted phosphorylation sites acted upon by calcineurin and mutated in the Crz1^7S-A^ strain; the green and red boxes show the PolyQ domain and the zinc finger domains, respectively. **(B)** The Crz1^WT^ (ECt3) and Crz1^7S-A^ (ECt335) strains were grown at 24°C and then shifted to 37°C for 15 minutes prior to fixation with 4% formaldehyde and visualized by direct fluorescence microscopy. GFP-Nop1 served as a nucleolar marker. **(C)** Quantification of cells with Crz1 nuclear localization at 24°C and after thermal stress (37°C) for 15 minutes. Error bars represent standard deviation; quantifications were conducted using three biological replicates. **(D)** To assay Crz1 gel migration mobility, the Crz1^WT^ (ECt3), Crz1^4S-A^ (ECt223), Crz1^6S-A^ (ECt231) and Crz1^7S-A^ (ECt335) strains were grown to log phase at 24°C and then shifted to 37°C for 1 hr in the presence or absence of 1 μg/ml FK506. Protein was extracted and analysed by western blot with mCherry antibody. **(E)** Expression levels of the *CHS6* gene in WT (H99), *crz1*Δ (AFA3-3), Crz1^WT^, Crz1^6S-A^, and Crz1^7S-A^ strains employed in panel 3D were compared. Strains were grown at 24°C in YPD medium with or without 1 μg/ml FK506. RNA was isolated and the expression of the *CHS6* gene was assessed by real-time PCR. Results shown are representative of three biological replicates. **(F)** The wild-type (H99), *cna1*Δ (KK1), *crz1*Δ (AFA3-3), Crz1^7S-A^ (ECt335 and ECt362), and Crz1^WT^ (ECt3 and ECt4) strains were grown in YPD media. Five 10-fold serial dilutions of each strain were spotted onto YPD containing the indicated additives and incubated at 30°C for 48 hours. Cultures with no additives were incubated at 30°C or 37°C for 48 hours or 39°C for 72 hours. CR: Congo Red; CFW: calcofluor white.

To determine the role of calcineurin-dependent phosphosites in Crz1 function, we tested the effect of mutations in these phosphosites on Crz1 nuclear localization and transactivation activity. Substitutions of serine with the non phosphorylatable amino acid alanine were introduced in transgenic Crz1 constructs, which then were fused at their C-termini to the mCherry fluorescent protein and integrated into the genome of the *crz1*Δ mutant. Because individual or concomitant substitution of S288 and S508, which are the Crz1 phosphosite residues originally identified by the phosphoscreen, with alanine resulted in only 23% nuclear localization of Crz1 (**S2C Fig**), we hypothesized the occurrence of additional calcineurin-dependent phosphosites. Therefore, we performed an independent phosphoscreen analysis of the immunoprecipitated Crz1-mCherry protein from cells shifted from 25°C to 37°C in the absence or the presence of FK506. This analysis revealed a total of 12 calcineurin-dependent phospho-serine residues (including the above S288 and S508 residues originally identified) (**S2A and S2B Fig**), which were systematically mutated in various combinations. Combined substitution of 3 (S563, S565, S569), 4 (S288, S291, S294, S298) and (S288, S329, S508, S569 referred as Crz1^4S-A^ in **Fig 3C and 3D**), or 6 (S288, S329, S508, S569, S765, S810, referred as Crz1^6S-A^ mutant in **Fig 3C–3E**), serine residues to alanine, all resulted in about 20% Crz1 nuclear localization at 24°C (**S2C Fig and Fig 3B and 3C**). Strikingly, concurrent substitution of the seven serine residues (S103, S288, S329, S508, S569, S765, S810) with alanine (depicted in **Fig 3A and** referred to as Crz1^7S-A^ mutant in **Fig 3B–3F**) elicited Crz1 nuclear localization in 81% of the cells observed at 24°C. In contrast, at 24°C, only 5.09% of Crz1^WT^ cells exhibited Crz1 nuclear localization, and the majority of cells displayed diffuse cytoplasmic localization of Crz1 (**Fig 3B and 3C**).

Comparing the mobility shift of the recombinant Crz1 proteins to wild-type Crz1 when calcineurin is inhibited, we observed that concurrent mutations of four, six, or seven predicted sites did not result in complete abolishment of the mobility shift. However, at 37°C the reduction in the mobility shift of the Crz1^7S-A^ mutant is more evident in comparison to the Crz1^WT^ strain and remarkably further addition of FK506 did not alter the mobility of the major slowest migrating protein fraction (**Fig 3D**). Moreover, the Crz1^7S-A^ mutant exhibited stronger induction in the expression of the Crz1-dependent *CHS6* gene at 24°C compared to the induction shown by the WT strains, and remarkably this induction was partially resistant to FK506 (**Fig 3E**). Despite the robust nuclear localization and transactivation activity of the Crz1^7S-A^ mutant, when the phenotypes of the Crz1^WT^ and Crz1^7S-A^ mutants were compared, we did not observe differences in growth during thermal stress or on media containing 0.4 M calcium or cell-wall damaging agents (**Fig 3F).** Therefore, increased Crz1 nuclear localization alone is not sufficient to improve stress resistance.

Taken together, this data demonstrates that dephosphorylation of Crz1 at S103 in combination with the other 6 serine residues mutated in the Crz1^7S-A^ is critical to elicit Crz1 nuclear localization and transactivation activity resistant to FK506. These results also suggest that in addition to those mutated in the Crz1^7S-A^ mutant, other sites are acted upon by calcineurin, most likely S563 and S565, which are important to trigger complete Crz1 nuclear localization, or that another phosphatase may be acting in parallel with calcineurin.

### Pbp1 is a potential calcineurin target

**Fig 4:**
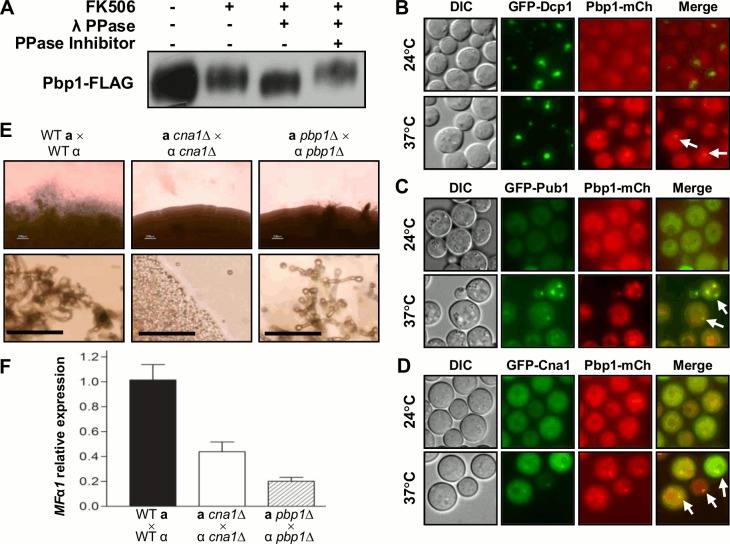
Calcineurin promotes sexual reproduction via Pbp1. **(A)** Pbp1-FLAG was immunoprecipitated from WT (HP181), unexposed or 1 μg/ml FK506 exposed cells grown at 24°C or shifted from 24°C to 37°C for 1 hr, and treated with λ phosphatase and/or phosphatase inhibitor *in vitro*. **(B-D)** Pbp1-mCherry co-localized with the PBs component Dcp1 (HP114) **(B)**, SGs component Pub1 (HP311) **(C)**, or Cna1 (HP268) **(D)** during a temperature shift to 37°C. Each strain was grown at 24°C or shifted from 24°C to 37°C for 1 hr and visualized with a DeltaVision Elite Deconvolution microscope. Arrows indicate the co-localization of Pbp1-mCherry with Dcp1 **(B)**, Pub1 **(C)**, or Cna1 **(D)**. **(E)** For sexual reproduction assays, the WT (H99, KN99**a**), and the *cna1*Δ (HP242, HP243), *pbp1*Δ (HP6, HP246) mutant strains were co-cultured with the opposite mating type strain on V8 media and incubated at room temperature in the dark for 7 days. Cells and the periphery of colonies were visualized by microscopy (upper panel). The bottom panel shows higher magnification views of the edges of colonies (Scale bars = 50 μm). **(F)** Pheromone gene expression (*MFα1*) was analyzed in WT, *cna1*Δ, and *pbp1*Δ mutants. The WT (H99, KN99**a**), *cna1*Δ (HP242, HP243), and *pbp1*Δ (HP6, HP246) mutant strains were co-cultured with the opposite mating type cells on V8 media and incubated at room temperature in the dark for 24 hours. Cells were harvested, RNA was isolated, and expression of *MFα1* was assessed by real-time PCR and plotted as the mean of three independent determinations with standard deviation as error bars. Results shown in **Fig 4A–4E** are representative of two independent experiments.

In the budding yeast *S*. *cerevisiae* Pbp1 (Pab1-Binding Protein) localizes in PBs/SGs under stress conditions and is essential for RNA processing including polyadenylation, splicing, and degradation [47,58,59]. In our phosphoscreen, four Pbp1 phosphopeptides were more abundant in both FK506 treated and *cna1*Δ mutant cells **(Fig 1, S4 Table)**. To test whether Pbp1 is regulated by calcineurin, *in vitro* phosphatase assays were performed with Pbp1-FLAG protein immune-isolated from WT cells or WT cells treated with FK506. We observed that the Pbp1-FLAG protein exhibited increased mobility when treated with λ phosphatase and this mobility was reduced by treatment with phosphatase inhibitors **(Fig 4A)**, strongly suggesting that Pbp1 is a potential calcineurin substrate.

As calcineurin A mainly co-localizes with components of PBs/SGs at 37°C [39], we next examined the localization of Pbp1 under thermal-stress conditions. Pbp1-mCherry fluorescence was observed in cytosolic puncta at 24°C and upon shift to 37°C these Pbp1-mCherry puncta co-localized with the P-body marker Dcp1, **(Fig 4B)** or with the stress granule component Pub1 **(Fig 4C)**. Interestingly, Pbp1 also co-localized with the calcineurin catalytic subunit Cna1 in PBs/SGs in response to thermal stress **(Fig 4D)**.

In *S*. *cerevisiae*, Pbp1 is involved in the regulation of mating-type switching in mother cells by post-transcriptionally controlling expression of the *HO* endonuclease [60]. As calcineurin is necessary for hyphal elongation during sexual reproduction in *C*. *neoformans* [36], we tested whether Pbp1 is involved in this process. Both *cna1*Δ and *pbp1*Δ mutant strains exhibited sexual reproduction defects **(Fig 4E)** in bilateral mating assays. The defect in sexual reproduction caused by the *cna1* mutation was more severe than the one observed in the *pbp1* mutant. Moreover, similar to the *cna1* mutant strain, the *pbp1* mutant showed a marked decrease in pheromone gene (*MF*α*1*) expression levels during sexual reproduction **(Fig 4F)**, suggesting that Pbp1 is required for activation of pheromone gene expression or mRNA stability. Collectively, these results provide evidence that calcineurin positively regulates sexual reproduction in part by controlling the phosphorylation state of Pbp1.

### Mutation of calcineurin target proteins confers stress sensitivity and impairs sexual reproduction

**Fig 5:**
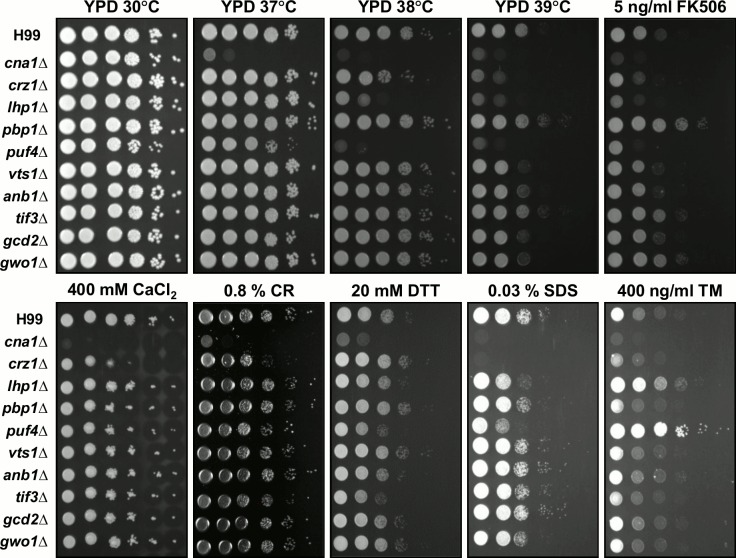
Phenotypes of the calcineurin target mutants exposed to various stresses. Spot dilution assays with WT (H99), *cna1*Δ (HP243), *crz1*Δ (HP235), *lhp1*Δ (HP22), *pbp1*Δ (HP6), *puf4*Δ (HP17), *vts1*Δ (HP24), *anb1*Δ (HP36), *tif3*Δ (HP9), *gcd2*Δ (HP28), and *gwo1*Δ (HP1) mutants were performed under several stress conditions as indicated. Strain cultures were incubated overnight, serially diluted 10-fold, and plated on YPD medium without or with FK506, CaCl_2_, Congo red (CR), dithiothreitol (DTT), sodium dodecyl sulfate (SDS), and tunicamycin (TM) at the indicated concentrations. Cells were incubated for 2 to 3 days at 30°C, 37°C, 38°C, or 39°C as indicated and all cultures with compound additions were incubated at 30°C. Results shown are representative of two independent experimental replicates.

Calcineurin controls responses to stress conditions, and we reasoned that calcineurin may promote survival during stress by dephosphorylating key targets. Mutants were generated in several of the identified potential calcineurin substrates, including Puf4, Pbp1, Vts1, Anb1, Gcd2, and Gwo1 **(Fig 1C),** and these mutants were then subjected to phenotypic analysis under a variety of stress conditions **(Fig 5)**. We confirmed previous reports that the *puf4*Δ mutant exhibits a pattern of sensitivity to multiple stresses, including heat, FK506, Congo red, SDS, or dithiothreitol (DTT), but in contrast to a prior study it was resistant to tunicamycin [61,62]. The *lhp1*Δ mutant exhibited increased sensitivity to heat, FK506, or SDS, whereas the *pbp1*Δ mutant showed more resistance to heat stress or FK506 as compared to the WT. Finally, mutation of *TIF3* caused an increased sensitivity to ER stress imposed by exposure to DTT **(Fig 5)**. Taken together, these results indicate that Puf4, Lhp1, Pbp1, and Tif3 contribute to stress survival.

**Fig 6:**
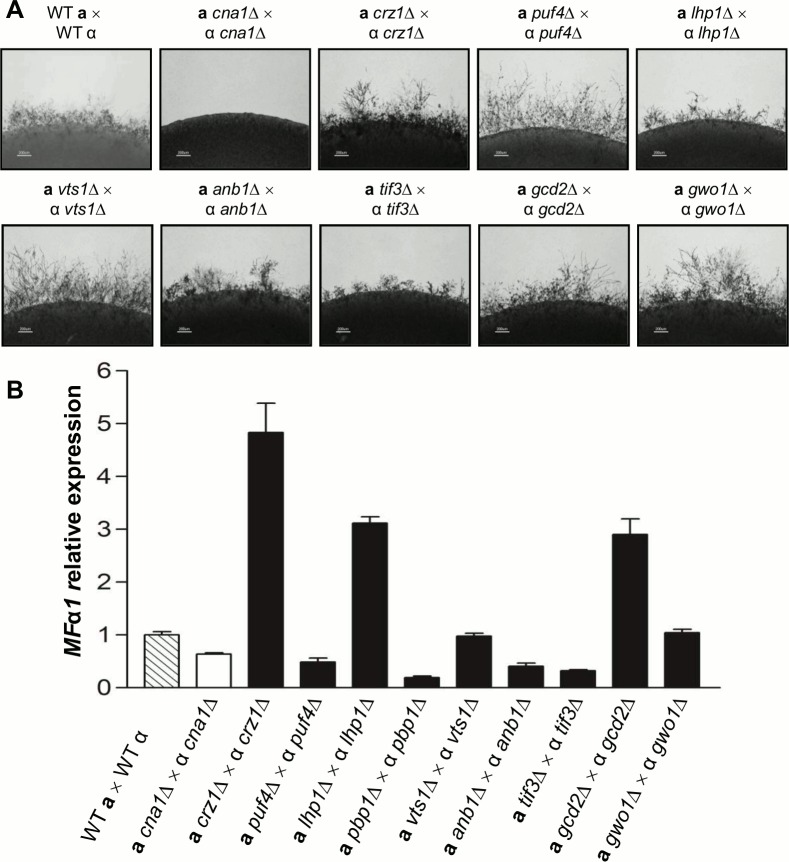
Sexual reproduction phenotypes of calcineurin target mutants. **(A)** Sexual reproduction assays for calcineurin target mutants were conducted. The WT (H99, KN99), and the *cna1*Δ (HP242, HP243), *crz1*Δ (HP235, HP239), *lhp1*Δ (HP22, HP258), *pbp1*Δ (HP6, HP246), *puf4*Δ (HP17, HP254), *vts1*Δ (HP24, HP261), *anb1*Δ (HP36, HP263), *tif3*Δ (HP9, HP250), *gcd2*Δ (HP28, HP264), and *gwo1*Δ (HP1, HP266) mutant strains were co-cultured with the opposite mating type strain on MS media and incubated at room temperature in the dark for 7 days. Several calcineurin targets including *LHP1*, *PUF4*, and *TIF3* are required for proper sexual reproduction. Results shown are representative of two independent experimental replicates. **(B)** Pheromone gene (*MF*α*1*) expression was analyzed in WT, *cna1*Δ, and the indicated calcineurin target mutants mating crosses following incubation in V8 media at room temperature for 24 hrs as described in the legend to **Fig 3C**. The results shown are the mean of three independent determinations with standard deviation as error bars.

In *C*. *neoformans*, calcineurin is also required for sexual reproduction, implying that calcineurin targets may also be involved in sexual development. To assess this, we tested the phenotypes conferred by mutating calcineurin targets and found that *tif3*Δ mutants, similar to *pbp1*Δ mutants, exhibit a defect in dikaryotic hyphal production **(Fig 6A)**. The *tif3* mutation also resulted in reduced expression levels of the pheromone gene (*MF*α*1*) during mating, suggesting that Tif3 is required for proper sexual reproduction. The *puf4*Δ mutants exhibited a hyper-filamentation phenotype and reduced expression levels of the pheromone gene **(Fig 6A and 6B).** In addition, *puf4*Δ mutants showed defective basidiospore formation, implying that Puf4 is essential for basidiospore chain formation during bisexual reproduction. These results suggest that in addition to Pbp1, Tif3 and Puf4 are also involved in sexual reproduction.

Calcineurin is relocalized from the bulk cytoplasm to PBs/SGs in response to heat and other stresses [39], and we hypothesize that a fraction of the calcineurin targets may co-localize with calcineurin in PBs/SGs. To test this hypothesis, we generated mCherry-tagged alleles of selected calcineurin target proteins known to exhibit RNA binding activity and examined their localization. We found that Puf4, Vts1, Tif3, and Gwo1-mCherry all re-localized to P-bodies following shift from 24°C to 37°C in *C*. *neoformans* **(S3 Fig)**. Other potential calcineurin substrates including Lhp1, Gcd2, and Anb1-mCherry did not co-localize with Dcp1. Taken together, we propose that calcineurin re-localizes to P-bodies and may regulate the function of potential targets at this location including Puf4, Pbp1, Vts1, Tif3, and Gwo1.

### Crz1 and other calcineurin targets function in a branched pathway

**Fig 7:**
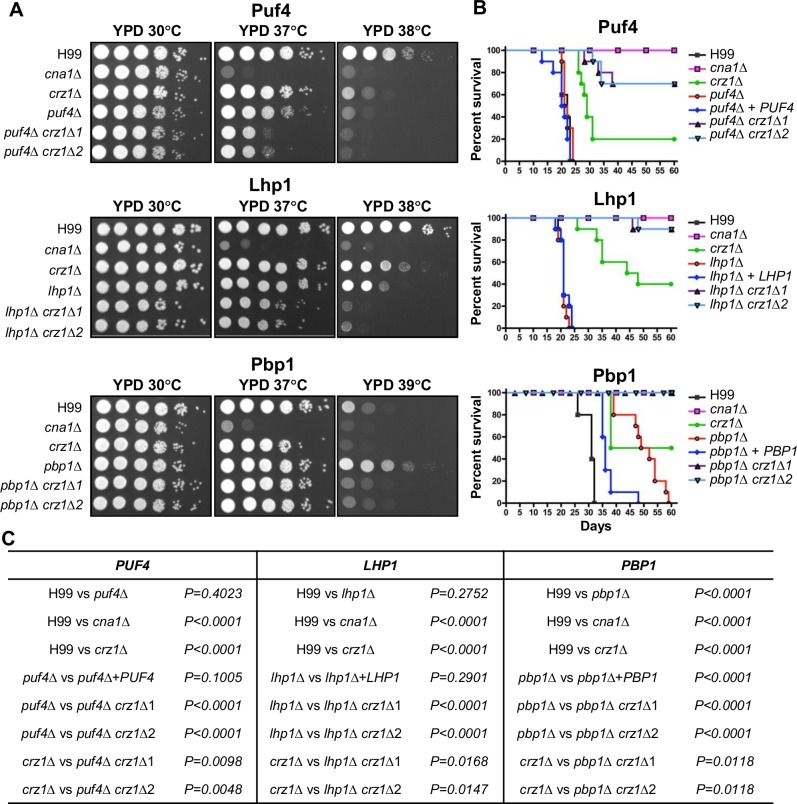
Crz1, Puf4, Lhp1, and Pbp1 collaborate to support growth at high temperature and virulence. **(A)** Thermosensitivity of (H99), the single *cna1*Δ (KK1), *crz1*Δ (LK343), *puf4*Δ (HP17), *lhp1*Δ (HP22), *pbp1*Δ (HP6), or double *puf4*Δ *crz1*Δ (HP163, HP164), *lhp1*Δ *crz1*Δ (HP166, HP167), and *pbp1*Δ *crz1*Δ (HP156, HP157) deletion mutant strains. Cells were grown overnight at 30°C, serially diluted 10-fold, and plated on YPD medium. Plates were incubated for 48 hours at 30°C, 37°C, 38°C, or 39°C as indicated. **(B)** Virulence of WT, single, or double deletion mutants listed in panel A. Cells were grown overnight in YPD liquid medium at 30°C, collected, washed with PBS, and 5 × 10^5^ (for Puf4 and Lhp1 experiments) or 5 × 10^4^ (for Pbp1 experiment) cells were inoculated into female A/J mice via intranasal instillation. Animals were sacrificed at defined endpoints of imminent mortality and survival was plotted. Animal survival was monitored for 60 days post infection. **(C)** p-value of virulence experiment shown in **Fig 5B**.

While Crz1 is a major calcineurin target, it contributes to but is not essential for survival at high temperature. Additionally, we found that mutation of three calcineurin targets, *pbp1*, *puf4*, and *lhp1*, conferred defects (*puf4*, *lhp1)* or enhanced (*pbp1*) thermotolerance **(Fig 5),** which were complemented by expression of the corresponding WT allele **(S4 Fig)**. Therefore, we hypothesize that the calcineurin network governing cell stress responses is branched with additional targets including Puf4, Lhp1, and Pbp1 that are regulated by calcineurin and function in parallel with Crz1. To test this model, we conducted epistasis analysis by generating double mutants and examining thermosensitivity and virulence phenotypes. As presented above, the *pbp1*Δ mutant, unlike the *puf4*Δ and *lhp1*Δ mutants, is resistant to heat stress **(Fig 5)**. Genetic analysis revealed that the *pbp1*Δ *crz1*Δ double mutant exhibited phenotypes that are intermediate between the *pbp1*Δ and *crz1*Δ single mutant phenotypes **(Fig 7A)**, suggesting that Crz1 and Pbp1 play opposite roles in heat stress responses. We also found that growth of the *puf4*Δ *crz1*Δ or *lhp1*Δ *crz1*Δ double mutant strains was more severely impaired at 37°C or 38°C compared to either the *crz1*Δ or the *lhp1*Δ and *puf4*Δ single mutant strains **(Fig 7A)**. These results demonstrate that Crz1 and Lhp1, and Crz1 and Puf4 together play additive roles in thermoresistance. Overall, these results validate that Crz1 functions in pathways parallel to the RNA binding proteins Puf4, Lhp1, and Pbp1 in responses to heat stress.

Virulence tests were conducted assessing the single and double mutants in the murine inhalation model **(Fig 7B and 7C).** First, as reference we found that the *crz1*Δ mutant shows attenuated virulence compared to WT, but not to the same degree as the *cna1*Δ mutant, which is avirulent. Second, mutation of *PUF4* alone did not result in a virulence defect under these conditions. In an insect larval model, however, the *puf4*Δ mutant was attenuated compared to the wild type strain **(S5A Fig)**. In addition, when mice were infected via intranasal instillation with a ten-fold lower inoculum (5×10^4^ CFU), *puf4*Δ mutants were very modestly attenuated compared to the wild type and complemented strains, and *crz1*Δ *puf4*Δ double mutants were avirulent up to 60 days post-infection and thus more attenuated than either single mutant alone **(S5B Fig)**. Third, mutation of *LHP1* alone did not result in a virulence defect, whereas *lhp1*Δ *crz1*Δ double mutant strains were attenuated compared to the *lhp1*Δ and *crz1*Δ single mutants **(Fig 7B)**. Fourth, the *pbp1*Δ single mutant strain showed attenuated virulence in a murine model and the *pbp1*Δ *crz1*Δ double mutant was avirulent in the murine inhalation model compared to the partial attenuation of either single mutant parent **(Fig 7B)**. The phenotypes of the *crz1* mutation combined with the *puf4*, *lhp1*, and *pbp1* mutations are additive, and this supports a model in which Puf4, Lhp1, and Pbp1 operate in a pathway parallel to Crz1. These findings are consistent with models whereby Crz1 and the RNA binding proteins Puf4, Lhp1, and Pbp1 function in a branched pathway controlled by calcineurin to promote high temperature growth and virulence.

## Discussion

In this study, we employed a phospho-proteomic approach and identified calcineurin targets that are associated with thermal stress responses and virulence of *C*. *neoformans*. More targets where identified when calcineurin was inactivated by mutation than by drug inhibition with FK506, and we attribute this to permanent in opposed to transient inhibition effects. However, irrespective of the differences in the phosphorylation profiles perturbed by calcineurin mutation versus inhibition, which are only partially overlapping, either mutation or inhibition of calcineurin is fully sufficient to render *Cryptococcus neoformans* sensitive to temperature and to other stress conditions that occur in the host. These observations fully validate calcineurin as an antifungal drug target. One of the key calcineurin targets identified is the *Cryptococcus* ortholog of the calcineurin-activated zinc finger transcription factor Crz1. Crz1 is a *bona fide* calcineurin target in yeasts and other ascomycetous fungi, but because of limited sequence homology conservation, whether or not it is conserved in basidiomycetous fungi had been unclear. It also had been unclear whether the identified Crz1 ortholog is a direct calcineurin target in basidiomycetes. Two conflicting results on Crz1 have been reported in *C*. *neoformans* [33,34]. Adler *et al*. reported that GFP-Crz1 constitutively localizes in the nucleus, suggesting that Crz1 is not a calcineurin substrate [34]. Djordjevic and colleagues found that localization of Crz1-GFP is calcineurin-dependent and proposed that Crz1 may be a calcineurin target; however, neither study addressed whether Crz1 is a direct calcineurin substrate [33]. Here we demonstrate that dephosphorylation, nuclear localization, and transcriptional activity of Crz1 are directly dependent upon calcineurin **(Figs 2 and 3)**.

Other calcineurin targets identified in our study include the RNA-binding proteins Lhp1, Puf4, and Pbp1. These RNA-binding proteins recognize and bind specific RNA sequences or structures to control multiple processes such as mRNA localization, stability, and function [63]. Among these RNA-binding proteins, Puf4 and Pbp1 together with Cna1 are concentrated in PBs/SGs in response to a shift to high temperature growth, suggesting that re-localization of calcineurin is a critical process for interaction with substrates [39]. In contrast, at either 24°C or 37°C, Lhp1-mCherry localizes to large cytoplasmic puncta that do not co-localize with Dcp1 or Cna1, suggesting that Lhp1 is either an indirect target of calcineurin or that calcineurin that has not been re-localized to PBs/SGs acts on Lhp1. However, with the exception of Pbp1, the remaining RNA binding proteins, including Lhp1, Puf3, Vts1, Anb1, Tif3, Gcd2, and Gwo1, should be considered as candidate calcineurin targets until they be subjected to further phosphorylation studies.

The Puf proteins are members of a conserved family of sequence-specific RNA binding proteins that bind to the 5’ or 3’ UTRs of mRNA transcripts in eukaryotes [64,65]. This protein family controls translation of specific mRNA targets [66]. Recently, Lee and colleagues found that phosphorylation of Puf3 controls the translation of Puf3 target mRNAs that are associated with mitochondrial biogenesis in *S*. *cerevisiae* [67]. In *C*. *neoformans*, Puf4 is required for splicing and decay of the mRNA encoding the unfolded protein response transactivator Hxl1 [61]. Thus, mutation of *PUF4* results in delayed splicing and expression of *HXL1* mRNA in response to temperature or ER stresses. Interestingly, calcineurin is also involved in thermotolerance mediated by the unfolded protein response (UPR) [68]. Thus, we considered the intriguing possibility that calcineurin controls *HXL1* expression via Puf4 phosphorylation and thereby contributes to responses to ER stress. However, in contrast to a previous report [61] and to the tunicamycin hypersensitivity exhibited by the *cna1* mutant, *puf4* mutation did not alter sensitivity to tunicamycin. However, it is possible that calcineurin and Puf4 are involved in the UPR via independent pathways. In agreement with previous studies, the *puf4*Δ mutant strain exhibited a similar degree of virulence to that seen with the WT in a murine infection model **(S5A Fig)**. However, our results showed that *puf4*Δ mutants were very modestly attenuated compared to wild type in insect larval and mouse models **(S5B Fig)**. The *puf4*Δ *crz1*Δ double mutant was more attenuated than either single mutant in either mice or insects **(S5 Fig)**. These results support the conclusion that Puf4 contributes to the virulence of *Cryptococcus*.

While calcineurin is globally conserved, previous studies have proposed that calcineurin targets are remarkably different across divergent fungal species [46]. We found that the calcineurin targets identified in this study share little overlap with known calcineurin substrates in *S*. *cerevisiae*, with the exception of Crz1. In particular, the mRNA binding proteins Lhp1, Puf4 and Pbp1 comprise a cohort of novel potential calcineurin targets, which have not been previously linked to calcineurin in mammals or in *S. cerevisiae*. Thus, while calcineurin dephosphorylates different targets in different fungi, calcineurin plays a similar role in stress survival in *S*. *cerevisiae* and *C*. *neoformans*, but a unique role in virulence of *C*. *neoformans* and other pathogenic fungi. Taken together, our results suggest that calcineurin cellular networks have been extensively rewired throughout the evolution of ascomycetes and basidiomycetes from their last common ancestor. However, further analysis identifying calcineurin targets in other fungal species will be required to test this model.

**Fig 8:**
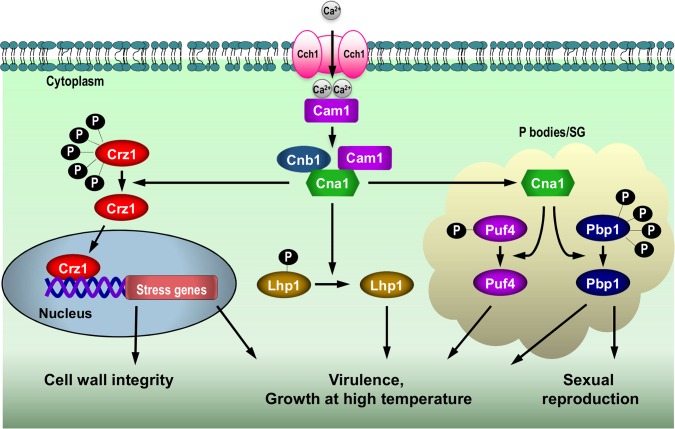
The calcineurin pathway controls growth at 37°C, virulence, and sexual reproduction via multiple targets. The calcineurin signaling network is branched to control distinct targets including Crz1, Lhp1, Puf4, and Pbp1 in different intracellular localizations (nucleus and PB/SGs) to evoke transcriptional and post-transcriptional programs necessary for *Cryptococcus* thermotolerance, virulence, and sexual reproduction (see Discussion).

In summary, our results support a model whereby activated calcineurin functions in a branched pathway to activate transcriptional and post-transcriptional processes that coordinate stress survival, virulence, and sexual reproduction **(Fig 8)**. At the transcriptional level, calcineurin dephosphorylates Crz1, which in turn, translocates into the nucleus to activate mRNA expression of genes linked to cell wall integrity, stress responses, and virulence. Second, calcineurin translocates into PBs/SGs where it targets post-transcriptional processes via Pbp1 and Puf4 to control thermotolerance, virulence, and sexual reproduction.

## Materials and Methods

### Strains, media, and growth conditions

Strains used in this study were derived from *C*. *neoformans* laboratory reference strain H99 and are listed in **S6 Table**. *C*. *neoformans* strains were maintained on YPD agar (1% yeast extract, 2% bactopeptone, and 2% dextrose) supplemented with the relevant antibiotics and grown at 30°C unless otherwise indicated.

For analysis of cell viability at different temperatures, 5 μl of 10-fold serial dilutions from overnight cultures were spotted on YPD agar (BD Difco) and incubated at a range of temperatures (30, 37, 38 and 39°C). To test stress-related phenotypes, 2.5 to 10 μl of cell cultures that had been grown in liquid YPD medium overnight were 10-fold serially diluted and spotted on YPD medium containing the indicated concentrations (see Fig legends) of the following compounds: calcium chloride, Congo red, tunicamycin, DTT (Sigma), FK506 (Astellas Pharma), and SDS (Teknova).

### Generation of mutant strains

Gene disruption cassettes were generated using an overlap-PCR strategy as previously described [69]. The oligonucleotides used in this study are listed in **S7 Table**. The 5’ and 3’-flanking regions of the target genes were amplified using primers 5F and 5R and primers 3F and 3R, respectively, and *C*. *neoformans* H99 genomic DNA as a template. Primers M13F and M13R were used to amplify the selectable markers conferring resistance to nourseothricin (NAT1) (Werner BioAgents) and G418 (NEO) (Gold Biotechnology) [70]. The final constructs for the gene disruption cassettes were generated by means of overlap PCR performed using primers NF and NR and the 5’ and 3’-flanking regions and markers as templates. The amplified gene disruption cassettes were purified using the QIAquick Gel Extraction kit (Qiagen) and then combined with gold microcarrier beads (Bio-Rad) and introduced into the H99 or *crz1*Δ mutant strains using the biolistic transformation method [71]. Multiple stable transformants were selected on YPD medium containing nourseothricin or G418, and then confirmed by diagnostic PCR for the 5’ and 3’ junctions followed by restriction enzyme digestion in each case.

To generate *MAT* **a** calcineurin-mutant strains, each *MAT*α calcineurin mutant and the WT strain KN99**a** were mixed in equal proportions on V8 media (pH = 5) and incubated in the dark at room temperature for 7 days. Basidiospores on the edges of a mating colony were removed and transferred onto YPD agar plates, and individual basidiospores were transferred onto a fresh YPD plate with G418 as described [72]. Isolated basidiospores were incubated at 30°C for 2 to 3 days and the resulting colonies were transferred to fresh YPD plates. Mating type determination was performed by PCR analysis using specific primers for *SXI1*α (*MAT*α; JOHE41440/JOHE41441) and *SXI2* **a** (*MAT* **a**; JOHE41442/JOHE41443) [73].

To generate the Pbp1-FLAG construct, the *PBP1* gene region including its predicted promoter was PCR amplified using the primer pair JOHE41154 and JOHE41155. The PCR product was then digested with NotI and cloned into pHP1, which contains a 4x FLAG tag, the terminator of the *HOG1* gene, and the hygromycin B-resistant gene. The resulting plasmid pHP4 was then introduced into the recipient *pbp1*Δ strains. The plasmids used in this study are listed in **S8 Table**.

### Generation of strains expressing mCherry-tagged calcineurin target proteins and Crz1-mCherry phosphosite mutants

The calcineurin target genes were replaced with calcineurin target-mCherry-NEO genes by homologous recombination as described previously [74]. Calcineurin target protein-mCherry chimera cassettes were generated using a modified overlap-PCR strategy. The 5’ (an ~1 kb region of the sequence immediately upstream of the start codon) and 3’ (an ~1 kb fragment of the sequence immediately downstream of the stop codon) flanking regions of the target genes were amplified using primers 5CF and 5CR and primers 3CF and 3R, respectively. The mCherry-NEO cassette was PCR amplified from plasmid pLKB25 with primers JOHE40808 and JOHE40809. The final PCR constructs were amplified with primers CNF and NR and were introduced into strain H99.

To construct the Crz1-mCherry fused strains for the site-directed mutagenesis, the Crz1 ORF was PCR amplified and fused with the mCherry fusion protein by splice overlap PCR, cloned into pCR2.1-TOPO (Invitrogen) to yield plasmid pXW15 which was sequenced. The 7.96 kb BamHI Crz1-mCherry fusion fragment was subcloned into the BamHI site of the safe haven plasmid pSDMA25, to generate plasmid pEC13 which was sequenced. Site-directed mutations were introduced into Crz1 ORF (pXW15 was used as the template) by PCR mutagenesis and then subcloned into pEC13 using the Gibson Assembly Master-mix (NEB). Plasmid clones of each mutagenic Crz1 allele were sequenced to ensure they contained the mutations. The recombinant Crz1-mCherry plasmid clones were linearized using the restriction enzyme AscI, and the *crz1*Δ::*NAT* deletion mutant was biolistically transformed. Targeted integration of each construct at the safe heaven locus was confirmed by PCR amplification (5’ junction: JOHE40956/JOHE40957; 3’ junction: JOHE40958/JOHE41562). Tandem array integration was ruled out by PCR using inverse primers (JOHE41450/JOHE41451) that would only yield a product if two or more copies of the construct were tandemly integrated at the safe haven site. The *NAT* selectable marker in pSL04, which contains the nucleolar fluorescence marker GFP-Nop1, was replaced with the *HYG* selectable marker from pJAF15. The GFP-Nop1 construct was introduced into the recombinant Crz1-mCherry mutant strains by ectopic integration. All primers used are listed in **S8 Table**.

### Sexual reproduction and pheromone gene expression assays

To analyze sexual reproduction phenotypes, the *MAT*α WT (H99) or calcineurin-mutants and *MAT* **a** WT (KN99**a**) or calcineurin mutant strains were grown overnight in liquid YPD media, washed with sterile water, mixed at equal density of cells (1×10^8^), spotted on MS (Sigma) or V8 (pH = 5) solid media, and incubated in the dark for 7 to 14 days at room temperature.

To examine pheromone gene expression, the *MAT*α WT (H99) or mutants and strain KN99**a** were mixed at equal cell densities, spread on V8 (pH = 5) solid media, and incubated in the dark for 24 hour at room temperature. All samples were collected, quick-frozen, and stored at -80°C. Total RNA was isolated from each sample using Trizol reagent (Thermo). Complementary DNA was synthesized using the AffinityScript QPCR cDNA Synthesis Kit (Agilent). Quantitative real-time PCR was performed with each gene-specific primer set and Brilliant III Ultra-Fast SYBR QPCR Mix (Agilent) and using a StepOnePlus Real-Time PCR system (ABI).

### *In vivo* virulence assay

*Cryptococcus* strains were grown overnight in YPD medium at 30°C. The cells were collected, washed twice with sterile PBS, counted with a hemocytometer, and the final density was adjusted to 1×10^7^ CFU/ml. Groups of 10 female A/J mice (Jackson Labs or NCI/Charles River Laboratories, 16~20 g) per strain were anesthetized with pentobarbital (Lundbeck Inc. Deerfield, IL), and inoculated with 5 × 10^4^ or 5 × 10^5^ CFU in a volume of 50 μl via intranasal inhalation as previously described [75]. Survival was monitored daily, and moribund mice were sacrificed with CO_2_. Survival curves were generated according to the Kaplan-Meier method by the Prism 4.0 (GraphPad software) and statistical significance (*p* values) assessed with the log-rank test.

### Ethics statement

All experiments and animal care were conducted in accordance with the ethical guidelines of the Institutional Animal Care and Use Committee (IACUC) of Duke University Medical Center (DUMC). The DUMC IACUC approved all of the vertebrate studies under protocol number A245-13-09. Mice studies were conducted in the Division of Laboratory Animal Resources (DLAR) facilities at DUMC and animals were handled according to the guidelines defined by the United States Animal Welfare Act and in full compliance with the DUMC IACUC.

### Sample preparation for mass spectrometry

WT (H99) and *cna1*Δ (KK1) cells were grown in YPD at 25°C to an optical density OD_600_ = 0.8~1 (log phase). Next, the WT cell culture was divided in thirds whereas the *cna1* culture was split in half, and one culture for each strain was incubated at 25°C while the rest of the cultures were transferred to 37°C for 1 hour. For FK506 treatment, one of the WT cultures was exposed to 2 μg/ml FK506 for 15 minutes prior to and during the shift to 37°C. Note that all cultures were conducted with 2 biological replicates for each condition analyzed. Following 1 hr incubation, cells were treated with 6% TCA (Sigma) and incubated on ice for 30 minutes to stop metabolic activity. Cells were collected by centrifugation, washed twice with cold acetone, and dried under vacuum. The dried pellets were resuspended in 500 μl of lysis buffer (50 mM Tris-Cl pH 7.5, 7 M urea, 10 mM NaF, 10 mM p-nitrophenylphosphate, 10 mM NaP_2_O_4_, 10 mM glycerophosphate, 1X Roche protease inhibitor cocktail and 1 mM PMSF) using a mini-bead beater (BioSpec) for 8 cycles (60 sec homogenization with 2 min rest). Protein was quantitated by a Bradford assay (Bio-Rad). Cell lysates were centrifuged at 14,000 rpm for 15 min at 4°C, supernatants were recovered, and protein was quantitated by a Bradford assay (Bio-Rad). For trypsin digestions 1.0 mg of protein from each sample was diluted 4.67 fold with 50 mM ammonium bicarbonate (Sigma) to achieve a final urea concentration of 1.5 M. Samples were treated with 20 mM DTT for 30 min at 70°C and alkylated with 40 mM iodoacetamide for 45 min at room temperature. Trypsin digestion (at a 1:50 w/w) was allowed to occur for 18 hr at 37°C. Digested samples were then acidified and a 600 μg total protein aliquot was removed from each sample. The remainder of the sample (total of 3.49 mg) was pooled and divided into three 600 μg aliquots to access TiO_2_ enrichment reproducibility (Enrichment quality control (QC)) and analytical reproducibility (Analytical QC). Each sample was desalted using a 50 mg Sep-Pack C18 Solid-Phase Extraction (SPE) cartridge. Briefly, samples were loaded onto pre-equilibrated SPE samples in acidified digestion buffer and washed twice 1 ml with 0.1% trifluoroacetic acid (TFA) in water. Samples were eluted in 1 ml 80% acetonitrile (MeCN), 0.1% TFA, partially dried using vacuum centrifugation and then brought to complete dryness using lyophilization.

### Sample preparation for Crz1 phosphoproteomics

The *crz1*Δ + Crz1^WT^ strain (AFA3-3-3) was grown in liquid YPD media at 24°C overnight. Cells were divided into three aliquots and diluted with fresh media (50 mL each) to an optical density of OD_600_ = 0.3. Cultures were further incubated at 24°C until an optical density of OD_600_ = 0.8 was reached. To one aliquot, FK506 (1 mg/mL final concentration) was added incubated at 24°C for 30 minutes prior to the temperature shift to 37°C. Two cultures, including the FK506-treated aliquot, were transferred to shaking water bath equilibrated to 37°C, and incubated for 30 minutes, while the third culture was kept at 24°C. After the 30 minute incubation, the cells from each treatment condition were collected by centrifugation, and washed once with ice-cold water, and then twice with ice-cold lysis buffer (10 mM Tris/HCl, pH 7.5, 150 mM NaCl, 0.5 mM EDTA, 1 mM PMSF, 1% Triton X100, 1X Roche protease inhibitor). Cells were pelleted and snap-frozen at -80°C. Frozen pellet was thawed on ice, with 500 μL of lysis buffer and 700 μL of acid-washed glass beads added. Cells were lysed using a mini-bead beater for 10 cycles (90 seconds homogenization with 2 minutes rest intervals) and the cell lysates cleared by centrifugation at 3,000 rpm for 10 minutes, at 4°C. The cell lysates were then further cleared of membrane-associated material by a second round of centrifugation at 14,000 rpm for 20 minutes. The RFP-Trap® beads (Chromotek, Planegg, Germany) were equilibrated following manufacturer’s protocol. 30 μL aliquots of the equilibrated beads were added to the cell lysates, and the lysate + bead suspensions were incubated with constant rotation at 4°C for 2 hours. The beads were pelleted by centrifugation at 700 rpm for 1 minute, and the supernatant removed. The beads were washed twice with lysis buffer, twice with wash buffer (10 mM Tris/HCl, pH 7.5, 500 mM NaCl, 0.5 mM EDTA), and twice with lysis buffer without the protease inhibitor cocktail. The beads were resuspended in ice-cold PBS with 1X Roche protease inhibitor cocktail, and 1 mM PMSF added and submitted to the Duke Proteomic Core Facility for phosphopeptide enrichment and phosphosite determination.

### Phosphopeptide enrichment

Each sample was resuspended in 150 μl of 80% MeCN, 1% TFA, 1 M glycolic acid and 30 fmol/μg of pre-trypsin digested bovine alpha casein. TiO_2_ enrichments were performed on GL Sciences p200 TiO_2_ spin columns following the manufacturer’s directions. Eluents were acidified with ~6 μl of formic acid. Samples were then frozen and lyophilized. Prior to LC-MS analysis, samples were resuspended in 10 μl of 200 mM ammonium format, 10 mM sodium citrate. A separate Analytical quality control (QC sample) (performed in triplicate) was generated by removing 5 μl from each of the Enrichment QC samples.

### Quantitative analysis of *Cryptococcus* phosphoproteome

Quantitative two-dimensional liquid chromatography-tandem mass spectrometry (LC/LC-MS/MS) was performed on 5 μl (50%) of each enriched sample. The method used two-dimensional liquid chromatography in a high-low pH reversed phase/reversed phase configuration on a nanoAcquity UPLC system (Waters Corp) coupled to a Synapt G2 HDMS high resolution accurate mass tandem mass spectrometer (Waters Corp.) with nanoelectrospray ionization as previously described [76–78]. Peptides were first trapped at 2 μl/min at 97/3 v/v water/MeCN in 20 mM ammonium formate (pH = 10) on a 5 μm XBridge BEH130 C18 300 um × 50 mm column (Waters). A series of step-elutions of MeCN at 2 μl/min was used to elute peptides from the first dimension column. Three steps of 4.7%, 9.4%, and 30% MeCN were employed; these percentages were optimized for delivery of an approximately equal load to the second dimension column for each fraction. For the second dimension separation, the eluent from the first dimension was first diluted 10-fold online with 99.8/0.1/0.1 v/v/v water/MeCN/formic acid and trapped on a 5 μm Symmetry C18 180 μm × 20 mm trapping column (Waters). The second dimension separations were performed on a 1.7 μm Acquity BEH130 C18 75 μm x 150 mm column (Waters) using a linear gradient of 3 to 30% MeCN with 0.1% formic acid over 36 min, at a flow rate of 0.5 μl/min and column temperature of 35°C. Data collection on the Synapt G2 mass spectrometer was performed in a data-dependent acquisition (DDA) mode, using 0.6 second MS scans with three subsequent 0.3 MS/MS scans in Resolution mode. CID fragmentation settings were charge state dependent. A dynamic exclusion list of 120 sec was employed to increase unique MS/MS triggers. The total analysis cycle time for each sample injection was approximately 4 hours. Two additional DDA acquisitions were performed on the pooled samples operating in sensitivity mode (two with 0.3s MS/MS scans and two with 0.6s MS/MS scans).

Data was imported into Rosetta Elucidator v3.3 (Rosetta Biosoftware, Inc), and all LC/LC-MS runs were aligned based on the accurate mass and retention time of detected ions (“features”) using PeakTeller algorithm (Elucidator). The relative peptide abundance was calculated based on area-under-the-curve (AUC) of aligned features across all runs and LC-MS feature maps (phosphorylation profiles) were generated for each analyzed condition. The overall dataset had 720,304 quantified features, and high collision energy (peptide fragment) data was collected in 105,784 spectra for sequencing by database searching. This MS/MS data was searched against the *Cryptococcus neoformans* H99 (http://www.broadinstitute.org/annotation/genome/cryptococcus_neoformans/MultiHome.html) database (n = 6954 entries), which also contained a reversed-sequence “decoy” database for false positive rate determination. Included in the database searches were variable modifications on methionine (oxidation, indicated by a M[147.035]), Asn/Qln (deamidation, indicated by a N[115.0269] or Q[129.0426]), and Ser/Thr/Tyr (phosphorylation, indicated by a S[166.9984], T[181.014] or Y[243.0297]). Individual peptide scoring using a Mascot Ion Score of 13.8 resulted in a 1.0% peptide false discovery rate.

To assess the workflow and technical reproducibility of this study we used three different approaches. First, based on the intensity data for six quality control samples (QC) samples (spiked bovine albumin in each of the two duplicates, which subsequently were analyzed in three technical replicates) included in this analysis, we calculated an average coefficient variation (CV) of intensity of 40.4%. In the second approach, we accessed the variability in each phosphopeptide peak area intensities across the three technical replicates analyzed from the same sample. This resulted in an average relative standard deviation (RSD) of 24%. The third assessment was to calculate the average variability in all of the phosphopeptides peak area intensities across a pooled sample, which was subjected to three separate TiO_2_ enrichments. This resulted in an average RSD in phosphopeptide peak area of 24.2%. A 2-fold cutoff is suitable unless the %CV of the QC samples is substantially lower or higher than 36%. There was not a power calculation involved.

### *In vitro* phosphatase and calcineurin assay

The strains expressing Crz1-FLAG or Pbp1-FLAG were grown in YPD at 24°C to an optical density OD_600_ 0.6–0.8 with or without FK506 (1 μg/ml). The culture was divided into two halves, and one half was incubated at 24°C and the other half at 37°C for 1 hour. Cells were collected by centrifugation, rapidly chilled using dry ice, and disrupted in lysis buffer (50 mM Tris-HCl pH = 7.5, 150 NaCl, 0.5 mM EDTA, 0.5% Triton X-100 supplemented with protease inhibitor tablet (Roche) and phosphatase inhibitor cocktails (Thermo)) using a mini-bead beater for 10 cycles (90 sec homogenization with 2 min rest). Cell extracts were centrifuged for 15 min at 14.000×*g*, the supernatant was recovered and protein concentration was determined by employing the BioRad Bradford reagent. The Crz1-FLAG protein was immunoprecipitated from the supernatant by incubating with anti-FLAG M2 affinity gel beads (Sigma) according to the manufacturer’s instructions. The immunoprecipitates were collected by centrifugation and washed with PBS (Sigma) supplemented with protease inhibitors.

For lambda phosphatase assays, the Crz1-FLAG immunoprecipitates bound to beads were incubated with PMP buffer (50 mM HEPES pH 7.5, 100 mM NaCl, 2 mM DTT, 0.01% Brij 35, 1 mM MnCl_2_) and 400 units of lambda protein phosphatase (New England BioLabs) (with or without phosphatase inhibitor cocktail) for 1 hour at 30°C. For calcineurin assays, the Crz1-FLAG beads were incubated in PMP buffer containing 400 units of human calcineurin (Enzo Life Sciences Inc.) at 30°C for 1 hour. The Crz1-FLAG beads were resuspended in Laemmli sample buffer (Bio-Rad), boiled for 10 min, briefly centrifuged, and the supernatant was resolved by SDS-PAGE and transferred to PVDF membranes (Bio-Rad). Membranes were assayed by western blot employing mouse monoclonal anti-FLAG M2 antibodies (Sigma), followed by anti-mouse antibody conjugated to horseradish peroxidase, and ECL western blotting detection reagent (GE Healthcare).

### Microscopy

Images of sexual hyphae were captured by using a Nikon Eclipse E400 microscope equipped with a Nikon DXM1200F camera. For fluorescence imaging of cells, unless otherwise indicated, the cell suspension was placed on a slide containing a 2% agar patch and covered with a coverslip. Fluorescence images were obtained using Deltavision system (Olympus IX-71 base) equipped with a Coolsnap HQ2 high resolution SSD camera. Images were processed using FIJI software.

## Supporting Information

S1 Fig Consensus motifs from calcineurin-dependent phosphopeptides. **(A)** Consensus motif derived from 47 calcineurin dependent phosphopeptides with phosphoserine residues showing enrichment of proline and arginine at the +1 and -3 positions, respectively. **(B)** Calcineurin-dependent phosphopeptides with phosphothreonine residues (10 in total) do not exhibit any characteristic amino acid sequence. Consensus motifs were generated employing the PhosphoSitePlus software available at http://www.phosphosite.org.
(TIF)

S2 Fig Mutation of the calcineurin-dependent Crz1 phosphorylation sites result in enriched nuclear localization at 24°C. **(A)** The intensity values of the Crz1 phosphopeptides were normalized to the non-phosphorylated Crz1 peptide AGLGEGELNSQPTLDPR. **(B)** Schematic diagram of the Crz1 protein drawn to scale. The yellow and blue pins indicate the predicted calcineurin-dependent phosphorylation sites; the green and red boxes depict the PolyQ domain and the zinc finger binding domains, respectively. **(C)** Cells with Crz1 nuclear localization at 24°C and following thermal stress (37°C) for 15 minutes were quantified. The Crz1^WT^ (ECt172), Crz1^S288A^ (ECt172), Crz1^S288,508A^ (ECt181), Crz1^S103A^ (ECt40), Crz1^S329A^ (ECt175), Crz1^S288,291,294,298A^ (ECt178), and Crz1^S563,565,569A^ (ECt41) strains were grown at 24°C and then shifted to 37°C for 15 minutes before fixation with 4% formaldehyde and visualization by direct fluorescence microscopy. Error bars represent the average with standard deviation for three biological replicates.
(TIF)

S3 Fig Localization of calcineurin target proteins: Puf4-mCherry, Tif3-mCherry, Vts1-mCherry, and Gwo1-mCherry co-localize with the P-body component Dcp1. The Puf4-mCherry (HP130), Lhp1-mCherry (HP133), Vts1-mCherry (HP138), Anb1-mCherry (HP142), Tif3-mCherry (HP123), Gcd2-mCherry (HP140), and Gwo1-mCherry (XW250) epitope tagged strains were grown at 24°C or shifted from 24°C to 37°C for 1 hour and visualized with a DeltaVision Elite Deconvolution microscope. GFP-Dcp1 serves as the PB marker. Arrows indicate the co-localization of the mCherry tagged calcineurin targets with GFP-Dcp1. Results shown are representative of two independent experimental replicates.
(TIF)

S4 Fig Thermotolerance or thermosensitivity of *puf4*, *lhp1*, and *pbp1* mutants. Spot dilution assays with WT, *puf4*, *lhp1*, and *pbp1* mutants and complemented strains were conducted. WT (H99), *cna1*Δ (KK1), *puf4*Δ (HP17), *lhp1*Δ (HP22), *pbp1*Δ (HP6) and complemented ((*puf4*Δ + *PUF4* (HP184, HP185), *lhp1*Δ + *LHP1* (HP188, HP189), *pbp1*Δ + *PBP1* (HP181, HP182)) strains were grown on YPD medium at 30°C, incubated overnight, serially diluted 10-fold, and plated on YPD medium. Cells were incubated for 2 days at 30°C, 37°C, 38°C, or 39°C as indicated. Results shown are representative of three independent experimental replicates.
(TIF)

S5 Fig Puf4 and Crz1 collaborate to control virulence of *Cryptococcus*. **(A)** Galleria larvae (12 per strain) were infected with 4000 cells each of the WT (H99), *cna1*Δ (KK1), *crz1*Δ (LK343), *puf4*Δ (HP17), *puf4*Δ + *PUF4* (HP181) and *puf4*Δ *crz1*Δ (HP163) mutant strains. Infected larvae were incubated at 37°C. Survival was monitored daily for 12 days. (H99 vs *cna1*Δ, *P<0*.*0001*; H99 vs *crz1*Δ, *P<0*.*0001*; H99 vs *puf4*Δ, *P = 0*.*0125*; *puf4*Δ vs *puf4*Δ+*PUF4*, *P = 0*.*1128*; *puf4*Δ vs *puf4*Δ *crz1Δ*, *P = 0*.*0477*; *crz1Δ* vs *puf4*Δ *crz1Δ*, *P = 0*.*0335*). **(B)** The *puf4*Δ mutant exhibits attenuated virulence compared to WT and *puf4* + *PUF4* complemented strain. WT (H99), *cna1*Δ (KK1), *crz1*Δ (LK343), *puf4*Δ (HP17), *puf4*Δ + *PUF4* (HP181) and *puf4*Δ *crz1*Δ (HP163) mutant strains were grown overnight in YPD medium at 30°C. 5 × 10^4^ CFU of each strain was inoculated into female A/J mice via intranasal instillation. (H99 vs *cna1*Δ, *P<0*.*0001*; H99 vs *crz1*Δ, *P<0*.*0001*; H99 vs *puf4*Δ, *P<0*.*0001*; *puf4*Δ vs *puf4*Δ+*PUF4*, *P<0*.*0001*; *puf4*Δ vs *puf4*Δ *crz1Δ*, *P<0*.*0001*; *crz1Δ* vs *puf4*Δ *crz1Δ*, *P = 0*.*0628*).
(TIF)

S1 Table Abundance of Phosphopeptides identified in this study. (XLSX)

S2 Table Phosphopeptides exhibiting a two-fold or more induction or reduction following FK506 treatment at 37°C. (XLSX)

S3 Table Phosphopeptides exhibiting a two-fold or more induction or reduction in the *cna1Δ* mutant at 37°C versus in the WT at 37°C. (XLSX)

S4 Table Calcineurin-dependent phosphopeptides identified by the phosphoscreen. (XLSX)

S5 Table Calcineurin-dependent phosphoproteins containing a PIxIxIT consensus sequence. (XLSX)

S6 Table Strains used in this study. (DOCX)

S7 Table Oligonucleotides employed in this study. (DOCX)

S8 Table Plasmids used in this study. (DOCX)
