# Supplementary figures and images for: Calcineurin Targets Involved in Stress Survival and Fungal Virulence
Source: PLoS Pathog. 2016 Sep 9;12(9):e1005873. doi: 10.1371/journal.ppat.1005873 (PMC5017699; doi:10.1371/journal.ppat.1005873)

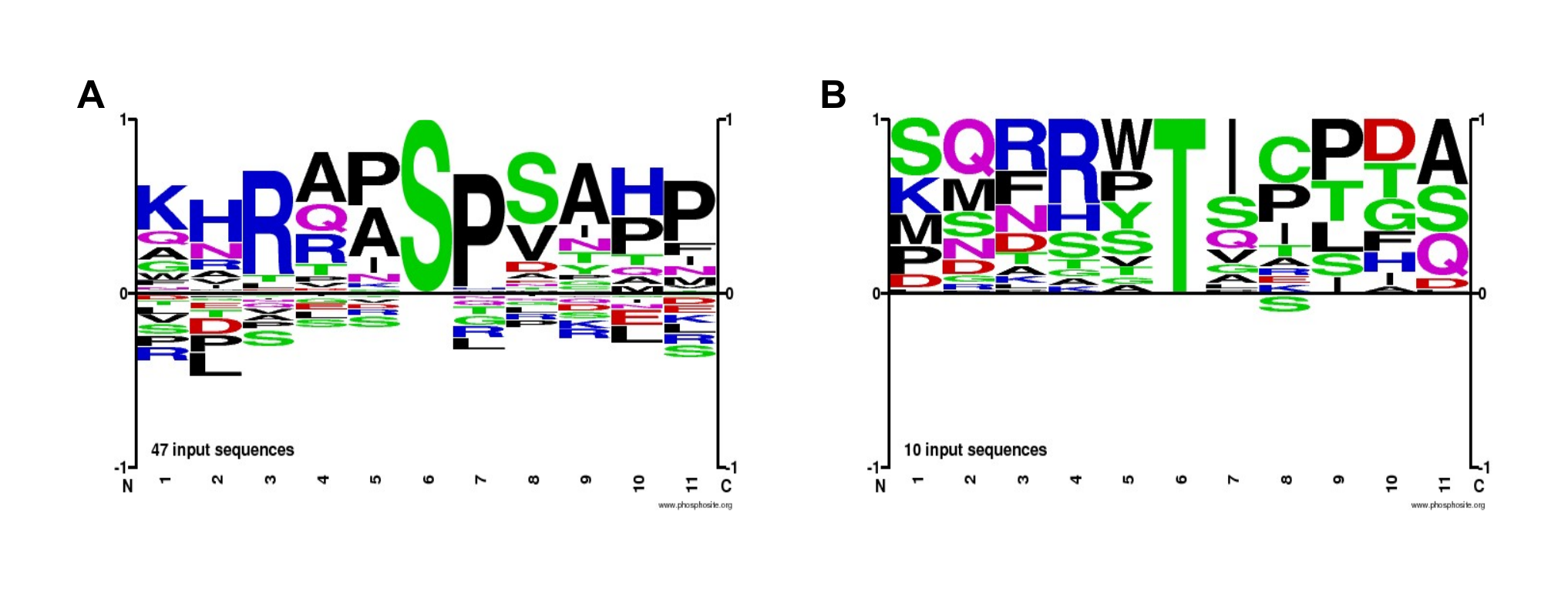

Supplement: S1 Fig — (A) Consensus motif derived from 47 calcineurin dependent phosphopeptides with phosphoserine residues showing enrichment of proline and arginine at the +1 and -3 positions, respectively. (B) Calcineurin-dependent phosphopeptides with phosphothreonine residues (10 in total) do not exhibit any characteristic amino acid sequence. Consensus motifs were generated employing the PhosphoSitePlus software available at http://www.phosphosite.org. (TIF) [file ppat.1005873.s001.tif]
