# Supplementary material for: Calcineurin Targets Involved in Stress Survival and Fungal Virulence
Source: PLoS Pathog. 2016 Sep 9;12(9):e1005873. doi: 10.1371/journal.ppat.1005873 (PMC5017699; doi:10.1371/journal.ppat.1005873)
Supplement: S6 Table — (DOCX) [file ppat.1005873.s011.docx]

**S6 Table. Strains used in this study.**

| **Strains** | **Relevant genotype** | **References** | **Figure** |
| --- | --- | --- | --- |
| H99 | MATalpha | [1] | 1, 2, 4B-C, 5-7, S4-5 |
| KN99 | MAT**a** | [2] | 4E-F, 6 |
| HP1 | MATalpha *gwo1*∆::NEO | This study | 5, 6 |
| HP6 | MATalpha *pbp1*∆::NEO | This study | 4E-F, 5, 6B, S4 |
| HP9 | MATalpha *tif3*∆::NEO | This study | 5, 6 |
| HP17 | MATalpha *puf4*∆::NEO | This study | 5, 6, S4, S5 |
| HP22 | MATalpha *lhp1*∆::NEO | This study | 5, 6, S4 |
| HP24 | MATalpha *vts1*∆::NEO | This study | 5, 6 |
| HP28 | MATalpha *gcd2*∆::NEO | This study | 5, 6 |
| HP36 | MATalpha *anb1*∆::NEO | This study | 5, 6 |
| HP64 | MATalpha *PBP1-mCherry*::NEO | This study | 4B-F |
| HP72 | MATalpha *TIF3-mCherry*::NEO | This study | S3 |
| HP80 | MATalpha *PUF4-mCherry*::NEO | This study | S3 |
| HP83 | MATalpha *LHP1-mCherry*::NEO | This study | S3 |
| HP84 | MATalpha *VTS1-mCherry*::NEO | This study | S3 |
| HP87 | MATalpha *GCD2-mCherry*::NEO | This study | S3 |
| HP95 | MATalpha *ANB1-mCherry*::NEO | This study | S3 |
| HP114 | MATalpha *PBP1-mCherry*::NEO *GFP-DCP1*::NAT | This study | 4B |
| HP123 | MATalpha *TIF3-mCherry*::NEO *GFP-DCP1*::NAT | This study | S3 |
| HP130 | MATalpha *PUF4-mCherry*::NEO *GFP-DCP1*::NAT | This study | S3 |
| HP133 | MATalpha *LHP1-mCherry*::NEO *GFP-DCP1*::NAT | This study | S3 |
| HP138 | MATalpha *VTS1-mCherry*::NEO *GFP-DCP1*::NAT | This study | S3 |
| HP140 | MATalpha *GCD2-mCherry*::NEO *GFP-DCP1*::NAT | This study | S3 |
| HP142 | MATalpha *ANB1-mCherry*::NEO *GFP-DCP1*::NAT | This study | S3 |
| HP156 | MATalpha *crz1*∆::NAT *pbp1*∆::NEO | This study | 7 |
| HP157 | MATalpha *crz1*∆::NAT *pbp1*∆::NEO | This study | 7 |
| HP163 | MATalpha *crz1*∆::NAT *puf4*∆::NEO | This study | 7 |
| HP164 | MATalpha *crz1*∆::NAT *puf4*∆::NEO | This study | 7 |
| HP166 | MATalpha *crz1*∆::NAT *lhp1*∆::NEO | This study | 7 |
| HP167 | MATalpha *crz1*∆::NAT *lhp1*∆::NEO | This study | 7 |
| HP181 | MATalpha *pbp1*∆::NEO *PBP1_4xFLAG*::HYG | This study | 4A, 7, S4 |
| HP182 | MATalpha *pbp1*∆::NEO *PBP1_4xFLAG*::HYG | This study | S4 |
| HP184 | MATalpha *puf4*∆::NEO *PUF4_4xFLAG*::HYG | This study | 7, S4, S5 |
| HP185 | MATalpha *puf4*∆::NEO *PUF4_4xFLAG*::HYG | This study | S4 |
| HP188 | MATalpha *lhp1*∆::NEO *LHP1_4xFLAG*::HYG | This study | 7, S4 |
| HP189 | MATalpha *lhp1*∆::NEO *LHP1_4xFLAG*::HYG | This study | S4 |
| HP235 | MATalpha *crz1*∆::NEO | This study | 6 |
| HP239 | MAT**a** *crz1*∆::NEO | This study | 6 |
| HP242 | MATalpha *cna1*∆::NEO | This study | 4E-F, 6 |
| HP243 | MAT**a** *cna1*∆::NEO | This study | 4E-F, 6 |
| HP246 | MAT**a** *pbp1*∆::NEO | This study | 4E-F, 6B |
| HP250 | MAT**a** *tif3*∆::NEO | This study | 6 |
| HP254 | MAT**a** *puf4*∆::NEO | This study | 6 |
| HP258 | MAT**a** *lhp1*∆::NEO | This study | 6 |
| HP261 | MAT**a** *vts1*∆::NEO | This study | 6 |
| HP263 | MAT**a** *anb1*∆::NEO | This study | 6 |
| HP264 | MAT**a** *gcd2*∆::NEO | This study | 6 |
| HP266 | MAT**a** *gwo1*∆::NEO | This study | 6 |
| HP268 | MATalpha *PBP1-mCherry*::NEO *GFP-CNA1*::NAT | This study | 4D |
| HP282 | MATalpha *CRZ1-mCherry*::NEO *GFP-NOP1*::NAT *cna1*∆::HYG | This study | 2E |
| HP289 | MATalpha *crz1*∆::NAT *CRZ1-4xFLAG*::NEO *cna1*∆::HYG | This study | 2C-D |
| HP311 | MATalpha *PBP1-mCherry*::NEO *GFP-PUB1*::NAT | This study | 4C |
| KK1 | MAT**a** *cna1*∆::NAT | [3] | 1, 2F, 7, S4, S5 |
| LK343 | MATalpha *crz1*∆::NAT | This study | 2F, 7, S5 |
| SEC435 | MATalpha *crz1*∆::NAT *CRZ1-4xFLAG*::NEO | This study | 2A-D |
| XW242 | MATalpha *GWO1-mCherry*::NEO | This study | S3 |
| XW250 | MATalpha *GWO1-mCherry*::NEO *GFP-DCP1*::NAT | This study | S3 |
| XW252 | MATalpha *CRZ1-mCherry*::NEO *GFP-NOP1*::NAT | This study | 2E |
| AFA3-3 | MATalpha *crz1*∆::NAT | This study | 3F |
| ECt3 | MATalpha *crz1*∆::NAT *CRZ1*^WT^-*mCherry*::NEO | This study | 3F |
| ECt4 | MATalpha *crz1*∆::NAT *CRZ1*^WT^-*mCherry*::NEO | This study | 3F |
| ECt172 | MATalpha *crz1*∆::NAT *CRZ1*^WT^-*mCherry*::NEO *GFP-NOP1*::HYG | This study | 3B |
| ECt40 | MATalpha *crz1*∆::NAT *CRZ1*^S103A^-*mCherry*::NEO *GFP-NOP1*::HYG | This study | S2 |
| ECt41 | MATalpha *crz1*∆::NAT *CRZ1*^S563, 565, 569A^-*mCherry*::NEO *GFP-NOP1*::HYG | This study | S2 |
| ECt49 | MATalpha *crz1*∆::NAT *CRZ1*^S288A^-*mCherry*::NEO *GFP-NOP1*::HYG | This study | S2 |
| ECt175 | MATalpha *crz1*∆::NAT *CRZ1*^S329A^-*mCherry*::NEO *GFP-NOP1*::HYG | This study | S2 |
| ECt178 | MATalpha *crz1*∆::NAT *CRZ1*^S288, 291,294, 298A^-*mCherry*::NEO *GFP-NOP1*::HYG | This study | S2 |
| ECt181 | MATalpha *crz1*∆::NAT *CRZ1*^S288, 508A^-*mCherry*::NEO *GFP-NOP1*::HYG | This study | S2 |
| ECt275 | MATalpha *crz1*∆::NAT *CRZ1*^4S-A^-*mCherry*::NEO *GFP-NOP1*::HYG | This study | 3C |
| ECt277 | MATalpha *crz1*∆::NAT *CRZ1*^6S-A^-*mCherry*::NEO *GFP-NOP1*::HYG | This study | 3B-E |
| ECt335 | MATalpha *crz1*∆::NAT *CRZ1*^7S-A^-*mCherry*::NEO *GFP-NOP1*::HYG | This study | 3B-F |
| ECt362 | MATalpha *crz1*∆::NAT *CRZ1*^7S-A^-*mCherry*::NEO | This study | 3F |

**References**

1. **Perfect JR, Lang SD, and Durack DT.** 1980. Chronic cryptococcal meningitis: a new experimental model in rabbits. Am. J. Pathol. 101**:**177-194

2. **Nielsen K., Cox GM, Wang P, Toffaletti DL, Perfect JR, and Heitman J.** 2003. Sexual cycle of Cryptococcus neoformans var. grubii and virulence of congenic ***a*** and α isolates. Infect. Immun. 71**:**4831-4841.

3. Kojima K, Bahn YS, and Heitman J. 2006. Calcineurin, Mpk1 and Hog1 MAPK pathways independently control fludioxonil antifungal sensitivity in *Cryptococcus neoformans*. Microbiology 152: 591–604.
