# Supplementary material for: Calcineurin Targets Involved in Stress Survival and Fungal Virulence
Source: PLoS Pathog. 2016 Sep 9;12(9):e1005873. doi: 10.1371/journal.ppat.1005873 (PMC5017699; doi:10.1371/journal.ppat.1005873)
Supplement: S7 Table — (DOCX) [file ppat.1005873.s012.docx]

**S7 Table. Oligonucleotides employed in this study.**

| **Name** | **Sequence (5’-3’)^a, b, c^** | **Purpose** |
| --- | --- | --- |
| **JOHE40706** | GTAAAACGACGGCCAG | *NAT*, *NEO*, *HYG* markers (M13F) |
| **JOHE40707** | CAGGAAACAGCTATGAC | *NAT*, *NEO*, *HYG* markers (M13R) |
| **JOHE40808** | ATGGTGAGCAAGGGCGAG | mCherry-*NEO* cassette |
| **JOHE40809** | CCAAGCTTGGTACCGAGCTC | mCherry-*NEO* cassette |
| **JOHE40708** | GGGCTACGTTTGGAATTAGCCTC | *CNA1* disruption (5F) |
| **JOHE40709** | CCCGCCATTACTTACGGTGTG | *CNA1* disruption (3R) |
| **JOHE40710** | TCACTGGCCGTCGTTTTAC GACGGAAATTGACTGTTTGGTG | *CNA1* disruption with marker (5R) |
| **JOHE40711** | CATGGTCATAGCTGTTTCCTG GAGTAGATGCTGGTTTCGAACG | *CNA1* disruption with marker (3F) |
| **JOHE40712** | GACCAGGATTCTCCCAATCTGC | *CNA1* disruption (NF_Nested) |
| **JOHE40713** | GTCATGGAGGCTCGCTACAAAG | *CNA1* disruption (NR_Nested) |
| **JOHE40714** | CCCATGTCGTTGTTCAGGTCAG | *GWO1* disruption (5F) |
| **JOHE40715** | GAAGACAATCATGCGATTGCGG | *GWO1* disruption (3R) |
| **JOHE40716** | TCACTGGCCGTCGTTTTAC CTAGGAGAGCGTTAGTATCTCACA | *GWO1* disruption with marker (5R) |
| **JOHE40717** | CATGGTCATAGCTGTTTCCTG GATGGCGGTGCAGAAAGCCA | *GWO1* disruption with marker (3F) |
| **JOHE40718** | CGTGATCTCACTGGAGCAGTTG | *GWO1* disruption (NF) |
| **JOHE40719** | GAAAGGGCACACCCTCAACC | *GWO1* disruption (NR) |
| **JOHE40726** | GCACCTGTCAAAGGCTCTTGG | *PBP1* disruption (5F) |
| **JOHE40727** | GTATCCGTTTCGGACCGACAG | *PBP1* disruption (3R) |
| **JOHE40728** | TCACTGGCCGTCGTTTTAC GCTGGGTCGTGACAGTCTAGGT | *PBP1* disruption with marker (5R) |
| **JOHE40729** | CATGGTCATAGCTGTTTCCTG TCGTTCGTCCTCTGATGTTAGC | *PBP1* disruption with marker (3F) |
| **JOHE40730** | CGGCATCCGCATCAGATCAC | *PBP1* disruption (NF) |
| **JOHE40731** | GTGTGGAGGACACATTGGTGG | *PBP1* disruption (NR) |
| **JOHE40799** | CGAAAGGAAATGCGTTCTTCCG | PBP1-mCherry construction (5CF) |
| **JOHE40800** | TCAACCCGTGGAAACATGGACC | PBP1-mCherry construction (CNF) |
| **JOHE40748** | CTCGCCCTTGCTCACCAT TCCTTGAGTCTGTCCGTTCTGC | PBP1-mCherry construction (5CR) |
| **JOHE40801** | GAGCTCGGTACCAAGCTTGG AAGTCGTTCGTCCTCTGATGTTAG | PBP1-mCherry construction (3CF) |
| **JOHE40738** | GGGAACGTACGAGGTCCTAACC | *TIF3* disruption (5F) |
| **JOHE40739** | CTTTGAGGGTGATCCTATCCCC | *TIF3* disruption (3R) |
| **JOHE40740** | TCACTGGCCGTCGTTTTAC ATGAATGTTAGGGTTGTAGGTGTA | *TIF3* disruption with marker (5R) |
| **JOHE40741** | CATGGTCATAGCTGTTTCCTG GGGAAAGCGTTAGTTATGTGTG | *TIF3* disruption with marker (3F) |
| **JOHE40742** | TGCTTCTTGTTCCTTGGGGTC | *TIF3* disruption (NF) |
| **JOHE40743** | TCTCTCCTTCAGGTAAACCTCC | *TIF3* disruption (NR) |
| **JOHE40805** | TGGAGGAGTAACAAGCCCTTAG | TIF3 -mCherry construction (5CF) |
| **JOHE40806** | ATCGCGACGTACCTCCTCACCA | TIF3 -mCherry construction (CNF) |
| **JOHE40752** | CTCGCCCTTGCTCACCAT AATCTTGACCTCCTCAACGCCC | TIF3 -mCherry construction (5CR) |
| **JOHE40807** | GAGCTCGGTACCAAGCTTGG AAAGAAAGGGAAAGCGTTAGTTAT | TIF3 -mCherry construction (3CF) |
| **JOHE40995** | AACTACGGCAAGGGCTCTCGTC | *PUF4* disruption (5F) |
| **JOHE40996** | AAGGAACGGAGCGGACTACCTG | *PUF4* disruption (3R) |
| **JOHE40997** | TCACTGGCCGTCGTTTTAC GTTTCGTAGTGTTCAAGGGTCAGAG | *PUF4* disruption with marker (5R) |
| **JOHE40998** | CATGGTCATAGCTGTTTCCTG ATGTCATCCCGCTGATCTTGTTTG | *PUF4* disruption with marker (3F) |
| **JOHE40999** | CTTATCCGGTAAGTTTGCTTCTG | *PUF4* disruption (NF) |
| **JOHE41000** | GAGCTTGTTCATCAGCCATGAAC | *PUF4* disruption (NR) |
| **JOHE41007** | CAGAGGATGCAGCTCGTCACC | PUF4 -mCherry construction (5CF) |
| **JOHE41008** | AGGTTCTCTGAGCCGCTCATC | PUF4 -mCherry construction (CNF) |
| **JOHE41009** | CTCGCCCTTGCTCACCAT CATCATCGGAACGTGGGAAGGC | PUF4 -mCherry construction (5CR) |
| **JOHE41010** | GAGCTCGGTACCAAGCTTGG GGAATGTCATCCCGCTGATCTTG | PUF4 -mCherry construction (3CF) |
| **JOHE41035** | GTCGCGCAAAGTGAATATCGC | *LHP1* disruption (5F) |
| **JOHE41036** | AGTCGAAACTGGTTTCGCCG | *LHP1* disruption (3R) |
| **JOHE41037** | TCACTGGCCGTCGTTTTAC GATGTCAAGAGGACAGTTGGCTAC | *LHP1* disruption with marker (5R) |
| **JOHE41038** | CATGGTCATAGCTGTTTCCTG TAGGGGTGTAATGGTGAGTGTTGGG | *LHP1* disruption with marker (3F) |
| **JOHE41039** | CTCCGGGCGACCTTTCTTCA | *LHP1* disruption (NF) |
| **JOHE41040** | ACGTTTCGCGTTCCCTCATTC | *LHP1* disruption (NR) |
| **JOHE41041** | AGGACACAAGGGACAGCTTGG | LHP1 -mCherry construction (5CF) |
| **JOHE41042** | ATGGGCATGTGCATGGACAAGG | LHP1 -mCherry construction (CNF) |
| **JOHE41043** | CTCGCCCTTGCTCACCAT CTGCTCATAGTTCTCTGACTCCTCG | LHP1 -mCherry construction (5CR) |
| **JOHE41044** | GAGCTCGGTACCAAGCTTGG TAGGGGTGTAATGGTGAGTGTTGG | LHP1 -mCherry construction (3CF) |
| **JOHE41045** | GTAGAGCATATGCGCAATGACTG | *VTS1* disruption (5F) |
| **JOHE41046** | CGGGGATGAGACTGGACTATTCG | *VTS1* disruption (3R) |
| **JOHE41047** | TCACTGGCCGTCGTTTTAC ATTGAGCCAGTTGTGTAGATTGACT | *VTS1* disruption with marker (5R) |
| **JOHE41048** | CATGGTCATAGCTGTTTCCTG ACTGCTCCCATCATCGGCCGCAT | *VTS1* disruption with marker (3F) |
| **JOHE41049** | GAAATGGCCTGCGTAGTGCATC | *VTS1* disruption (NF) |
| **JOHE41050** | CAGTGGTCTCAACATCCATTTC | *VTS1* disruption (NR) |
| **JOHE41051** | TGAAAGCGCTGCCTTCCCTC | VTS1 -mCherry construction (5CF) |
| **JOHE41052** | TCTGCTGGCAGCCCTGGTATTG | VTS1 -mCherry construction (CNF) |
| **JOHE41053** | CTCGCCCTTGCTCACCAT TTTGGATTCTTTGGGTCCGGGAG | VTS1 -mCherry construction (5CR) |
| **JOHE41054** | GAGCTCGGTACCAAGCTTGG ACTGCTCCCATCATCGGCCGCAT | VTS1 -mCherry construction (3CF) |
| **JOHE41055** | GGTGGAATTTGGAACGGACGG | *ANB1* disruption (5F) |
| **JOHE41056** | GCTATAGGTCGCAGATGGGATG | *ANB1* disruption (3R) |
| **JOHE41057** | TCACTGGCCGTCGTTTTAC TTCTAGAAATGGGTCAGCGTTGGAT | *ANB1* disruption with marker (5R) |
| **JOHE41058** | CATGGTCATAGCTGTTTCCTG GCGTCCCGCATACCTACCATCTTC | *ANB1* disruption with marker (3F) |
| **JOHE41059** | GTGAGGAGGAGATAACGGATGG | *ANB1* disruption (NF) |
| **JOHE41060** | GGCCTTCGATGAGAGTCTGTTAC | *ANB1* disruption (NR) |
| **JOHE41061** | GGTGTAATGGCCGTGACTTTGG | ANB1 -mCherry construction (5CF) |
| **JOHE41062** | TGAATGGGGCGTCACTCCTC | ANB1 -mCherry construction (CNF) |
| **JOHE41063** | CTCGCCCTTGCTCACCAT GTTGGCGGGAGCCTCCTTGTAG | ANB1 -mCherry construction (5CR) |
| **JOHE41064** | GAGCTCGGTACCAAGCTTGG GCGTCCCGCATACCTACCATCTTC | ANB1 -mCherry construction (3CF) |
| **JOHE41065** | CGTTGACGCATGAGGGTTTGG | *GCD2* disruption (5F) |
| **JOHE41066** | CCCTTCAAAAGGAGCTTGCCG | *GCD2* disruption (3R) |
| **JOHE41067** | TCACTGGCCGTCGTTTTAC TCGAATGGAATTGAGGCGACGATT | *GCD2* disruption with marker (5R) |
| **JOHE41068** | CATGGTCATAGCTGTTTCCTG CACGGTATGAATACGCAGTCCAG | *GCD2* disruption with marker (3F) |
| **JOHE41069** | CTGGATCTGTGAGGAGGTAATCC | *GCD2* disruption (NF) |
| **JOHE41070** | TGAGGCACCACGTTCAGGAGC | *GCD2* disruption (NR) |
| **JOHE41071** | CTCGAAACGGGCAAGATCCAC | GCD2 -mCherry construction (5CF) |
| **JOHE41072** | CCGCGTAGGTGTGCTGATGAAC | GCD2 -mCherry construction (CNF) |
| **JOHE41073** | CTCGCCCTTGCTCACCAT CACAACGCCACCGGATTTACCGA | GCD2 -mCherry construction (5CR) |
| **JOHE41074** | GAGCTCGGTACCAAGCTTGG TAGGATCCATTCATAATCATTATATATC | GCD2 -mCherry construction (3CF) |
| **JOHE41440** | CCAACAGAATGCGTCACCTC | Confirmation of *SXI1*α_F |
| **JOHE41441** | TTACTCCGATCTCTGCCCAC | Confirmation of *SXI1*α_F |
| **JOHE41442** | ACCAAAGCCCTCAGAGTCtg | Confirmation of *SXI2***a**_F |
| **JOHE41443** | GACCGGAAACCCCAACAGTA | Confirmation of *SXI2***a**_R |
| **JOHE41154** | atat **GCGGCCGC** GCATCCGCATCAGATCACCTTC | *PBP1*_Promoter_ORF_F |
| **JOHE41155** | atat **GCGGCCGC** TCCTTGAGTCTGTCCGTTCTGC | *PBP1*_Promoter_ORF_R |
| **JOHE41300** | atat **GCGGCCGC** AACTACGGCAAGGGCTCTCGT | *PUF4*_Promoter_ORF_F |
| **JOHE41301** | atat **GCGGCCGC** CATCATCGGAACGTGGGAAGG | *PUF4*_Promoter_ORF_R |
| **JOHE41303** | atat **GCGGCCGC** GTCGCGCAAAGTGAATATCGC | *LHP1*_Promoter_ORF_F |
| **JOHE41304** | atat **GCGGCCGC** CTGCTCATAGTTCTCTGACTCCTC | *LHP1*_Promoter_ORF_R |
| **JOHE41391** | GGTACTCACAACTGAGCCAGCAG | *CRZ1* disruption (5F) |
| **JOHE41392** | TCATCGTCGTCGAAGTCGAGGC | *CRZ1* disruption (3R) |
| **JOHE41393** | TCACTGGCCGTCGTTTTAC GTGGATTATAGGGGTGACTGATAGA | *CRZ1* disruption with marker (5R) |
| **JOHE41394** | CATGGTCATAGCTGTTTCCTG CGATGGTCATAGGGCGCTGTGAG | *CRZ1* disruption with marker (3F) |
| **JOHE41395** | GGTTCGTTAGTCGGGTCAACTG | *CRZ1* disruption (NF) |
| **JOHE41396** | TTAGGGGAGGTTGGGATCGG | *CRZ1* disruption (NR) |
| **JOHE41645** | aatt **GGATCC** TCCGTCGAAACCGCTACATCC | PUB1_ORF_F |
| **JOHE41646** | aatt **GGATCC** GAGGTCTTAAATGGGATGTCGG | PUB1_ORF_R |
| **JOHE42947** | TCTTGCGGCTCTCAATTATG | qPCR_*CHS6*_F |
| **JOHE42948** | CCTCAAGCTTATCGTCCACA | qPCR_*CHS6*_R |
| **JOHE42959** | CGCCTTCACTGCCATCTTCACC | qPCR_*MF*α*1*_F |
| **JOHE42960** | GCGATGACACAAAGGGTCATGC | qPCR_*MF*α*1*_R |
| **JOHE42966** | ACAATTGGCACAGATGTCGT | qPCR_*CHS5*_F |
| **JOHE42967** | ATGACAAACTTGTCGTGGGA | qPCR_*CHS5*_R |
| **JOHE42968** | TCGAAGTGGTCATGGTCCTA | qPCR_*CHS*7_F |
| **JOHE42969** | ATACCCATGCGAACACAAGA | qPCR_*CHS*7_R |
| **JOHE39093** | ATGGCAGATCCAGCCTCACCC | *CRZ1*-mCH construction (5CF) |
| **JOHE39078** | GGGGAGCAGATTGTGTATCTT | *CRZ1*-mCH construction (CNF) |
| **JOHE39079** | CTCGCCCTTGCTCACCATATCCTCTTCACTCGTTTCAC | *CRZ1*-mCH construction (5CR) |
| **JOHE39080** | GAGCTCGGTACCAAGCTTGGTCGCCCGATGGTCATAG | *CRZ1*-mCH construction (3CF) |
| **JOHE39082** | TGTGCTGAGGCTGTGTACCC | *CRZ1*-mCH construction (CNR) |
| **JOHE39098** | ATGTTTTACGTTTTAGGATA**g**CGAACACCTCCCACCCTT | Crz1 S288 to A (F) |
| **JOHE39099** | AAGGGTGGGAGGTGTTCG**c**TATCCTAAAACGTAAAACAT | Crz1 S288 to A (R) |
| **JOHE39143** | AAGACGGTTTCAACGTTCCT**g**CTCCACAATCCCATCCTTTAC | Crz1 S508 to A (F) |
| **JOHE39144** | GTAAAGGATGGGATTGTGGAG**c**AGGAACGTTGAAACCGTCTT | Crz1 S508 to A (R) |
| **JOHE40073** | AGGAGTCATTGAGAAAGGAG**g**CTCTGCCGTTCAATCCACAAGA | Crz1 S103 to A (F) |
| **JOHE40074** | TCTTGTGGATTGAACGGCAGAG**c**CTCCTTTCTCAATGACTCCT | Crz1 S103 to A (R) |
| **JOHE40083** | GGACAGAGTCTTTTCCTGCT**g**CACGATCGCCTAGCCCCTTTGC | Crz1 S329 to A (F) |
| **JOHE40084** | GCAAAGGGGCTAGGCGATCGTG**c**AGCAGGAAAAGACTCTGTCC | Crz1 S329 to A (R) |
| **JOHE40179** | ATGTTTTACGTTTTAGGATA**g**CGAACACC**g**CCCACCCT**g**CGCAAT  ATCCT**g**CGAGAACATCTTCACCTTTTC | Crz1 S288, S291, S294, S298 to A (F) |
| **JOHE40180** | GGAAAAGGTGAAGATGTTCTCG**c**AGGATATTGCG**c**AGGGTGGG**c**GG  TGTTCG**c**TATCCTAAAACGTAAAACAT | Crz1 S288, S291, S294, S298 to A (R) |
| **JOHE40181** | CGAGTCGCCCACGAGCCAAA**g**CTGAT**g**CCATCATTCCT**g**CTCCC  ACAGCCGATTCATTTGA | Crz1 S563, S565, S569 to A (F) |
| **JOHE40182** | TCAAATGAATCGGCTGTGGGAG**c**AGGAATGATGG**c**ATCAG**c**TTT  GGCTCGTGGGCGACTCG | Crz1 S563, S565, S569 to A (R) |
| **JOHE41936** | CATGCAACTGTTCGTCGTCGC**gca**TTTGGCGGTGGGGAACA | Crz1 S765 to A (F) |
| **JOHE41937** | TGTTCCCCACCGCCAAA**tgc**GCGACGACGAACAGTTGCATG | Crz1 S765 to A (R) |
| **JOHE42257** | AGGCGAGCCGCGAGG**gc**CGAAGATTTTGGAAGAG | Crz1 S810 to A (F) |
| **JOHE42258** | CTCTTCCAAAATCTTCG**gc**CCTCGCGGCTCGCCT | Crz1 S810 to A (R) |
| **JOHE40662** | GCCCACGAGCCAAATCTGAT**g**CCATCATTCCTTCTCCCACAGC | Crz1 S569 to A (F) |
| **JOHE40663** | GCTGTGGGAGAAGGAATGATGG**c**ATCAGATTTGGCTCGTGGGC | Crz1 S569 to A (R) |
| **JOHE39136** | GTCCACTATGCCAGCGTTCA | Crz1-mCH construct sequencing (1F) |
| **JOHE39137** | CCTGCCTTCCTTCAATCCAC | Crz1-mCH construct sequencing (2R) |
| **JOHE39138** | ATTCCCACCGTCAAATCCAG | Crz1-mCH construct sequencing (3F) |
| **JOHE39139** | GCTGATACAGTGCCGGACAA | Crz1-mCH construct sequencing (4R) |
| **JOHE39140** | GCAGCAGACTGGACAAATGC | Crz1-mCH construct sequencing (5F) |
| **JOHE39141** | GGCGTCTGCTGCTCTTCATA | Crz1-mCH construct sequencing (6R) |
| **JOHE39142** | GCCAGGCAACATGATTGTAAA | Crz1-mCH construct sequencing (7F) |
| **JOHE40141** | CAATAATAAGCGATATTTAG | Crz1-mCH construct sequencing (8F) |
| **JOHE40142** | GGACATTATCTTCAGATCAG | Crz1-mCH construct sequencing (9F) |
| **JOHE40684** | GGTTCTGCGGGAATAGAT | Crz1-mCH construct sequencing (10F) |
| **JOHE40685** | CACCTTTTCCTCAGCAATCA | Crz1-mCH construct sequencing (11F) |
| **JOHE40686** | TTGACGGTGGGAATAATC | Crz1-mCH construct sequencing (12R) |
| **JOHE40687** | CAGCTATTCGCAAGGTATC | Crz1-mCH construct sequencing (13R) |
| **JOHE41346** | CGAGTGAAGAGGATATGGTG | Crz1-mCH construct sequencing (14F) |
| **JOHE41347** | TACATGAACTGAGGGGAC | Crz1-mCH construct sequencing (15R) |
| **JOHE41348** | GCCCCGTAATGCAGAAGA | Crz1-mCH construct sequencing (16F) |
| **JOHE41349** | CGTTGTGGGAGGTGATGT | Crz1-mCH construct sequencing (17R) |
| **JOHE41350** | CACCCCACTACCAAGCGAA | Crz1-mCH construct sequencing (18F) |
| **JOHE41351** | ATACTCTCAACACCAGCG | Crz1-mCH construct sequencing (19R) |
| **JOHE40956** | AACTATACGCAGCATGAACAT | Crz1-mCH complementation diagnostic (5F) |
| **JOHE40957** | ACTTTCTACCTGGTCTGCTTT | Crz1-mCH complementation diagnostic (3R) |
| **JOHE40958** | AATAAGGGCGACACGGAAATG | Crz1-mCH complementation diagnostic (5R) |
| **JOHE41562** | ATGTGGATGTGGAGGAAGA | Crz1-mCH complementation diagnostic (3F) |
| **JOHE41450** | GCCCATAGACTTCAAATCATG | Crz1-mCH complementation diagnostic (Tandem array F) |
| **JOHE41451** | GGCAAGGGAACAGACAGATAC | Crz1-mCH complementation diagnostic (Tandem array R) |

^a^ Underlined sequence is homologous to vector or cassette sequence.

^b^ Lowercase indicates linker sequence.

^c^ Bold sequence denotes restriction site sequence.

^d^Bold lower case sequence denotes nucleotide substations to create the desired mutation.

F, R: reverse and forward, respectively.
