# Supplementary material for: Calcineurin Targets Involved in Stress Survival and Fungal Virulence
Source: PLoS Pathog. 2016 Sep 9;12(9):e1005873. doi: 10.1371/journal.ppat.1005873 (PMC5017699; doi:10.1371/journal.ppat.1005873)
Supplement: S8 Table — (DOCX) [file ppat.1005873.s013.docx]

**S8 Table. Plasmids used in this study.**

| **Plasmid** | **Relevant genotype** | **References** |
| --- | --- | --- |
| pLKB39 | GFP-CNA1 NAT | [1] |
| pLKB88 | mCH-PUB1 HYG | [1] |
| pXW11 | GFP-DCP1 NAT | [2] |
| pSL04 | GFP-NOP1 NAT | [3] |
| pCN19 | AmpR NAT | [4] |
| pHP1 | AmpR 4xFLAG HYG | This study |
| pHP3 | GFP-PUB1 NAT | This study |
| pHP4 | PBP1-4xFLAG HYG | This study |
| pHP5 | PUF4-4xFLAG HYG | This study |
| pHP6 | LHP1-4xFLAG HYG | This study |
| pSDMA25 | Safe haven plasmid | [5] |
| pXW15 | CRZ1-mCH NEO | This study |
| pEC13 | CRZ1-mCH NEO | This study |
| pEC14 | CRZ1^S103A^-mCH NEO | This study |
| pEC16 | CRZ1^S329A^-mCH NEO | This study |
| pEC17 | CRZ1^S288A^-mCH NEO | This study |
| pEC19 | CRZ1^S288, 508A^-mCH NEO | This study |
| pEC20 | CRZ1^S563, 565, 569A^-mCH NEO | This study |
| pEC22 | CRZ1^S288, 291, 294, 298A^-mCH NEO | This study |
| pEC86 | CRZ1^S288, 329, 508, 569A^-mCH NEO | This study |
| pEC89 | CRZ1^S288, 329, 508, 569, 765, 810A^-mCH NEO | This study |
| pEC93 | CRZ1^S103, 288, 329, 508, 569, 765, 810A^-mCH NEO | This study |
| pEC28 | GFP-NOP1 HYG | This study |

**References**

# 1. Kozubowski L, Aboobakar EF, Cardenas ME, and Heitman J. 2011. Calcineurin colocalizes with P-bodies and stress granules during thermal stress in *Cryptococcus neoformans*. Eukaryot Cell. 10:1396-402

# 2. Wang X, Hsueh YP, Li W, Floyd A, Skalsky R, and Heitman J. 2010. Sex-induced silencing defends the genome of *Cryptococcus neoformans* via RNAi. Genes Dev. 24:2566-2582.

# 3. Lee SC and Heitman J. 2012. Function of *Cryptococcus neoformans KAR7* (*SEC66*) in karyogamy during unisexual and opposite-sex mating. Eukaryot Cell. 11:783-94

# 4. Price MS, Nichols CB, and Alspaugh JA. 2008. The *Cryptococcus neoformans* Rho-GDP dissociation inhibitor mediates intracellular survival and virulence. Infect. Immun. 76:5729–5737.

# 5. Arras, S.D.M, Chitty, J.L., Blake, K.L., Schulz, B.L., and Fraser, J.A. 2015. A genomic safe haven for mutant complementation in *Cryptococcus neoformans*. PLOS One 10(4):e0122916
